# Association between amoxicillin administration and outcomes in critically ill patients with acute kidney injury

**DOI:** 10.3389/fphar.2024.1409654

**Published:** 2024-07-15

**Authors:** Xinyao Luo, Weijian Zhou, Dingyuan Wan, Jing Peng, Ruoxi Liao, Baihai Su

**Affiliations:** ^1^ Department of Nephrology, Kidney Research Institute, West China Hospital, Sichuan University, Chengdu, China; ^2^ Department of Intensive Care Medicine, West China Hospital, Sichuan University, Chengdu, China

**Keywords:** acute kidney injury, amoxicillin, intensive care unit, mortality, acute kidney disease

## Abstract

**Purpose:**

This study assessed the effect of amoxicillin on outcomes in intensive care unit (ICU) patients with acute kidney injury (AKI), focusing on mortality rates and acute kidney disease (AKD) occurrence.

**Materials and Methods:**

We conducted a retrospective cohort analysis utilizing data from the Medical Information Mart for Intensive Care IV (MIMIC-IV) database. The study included intensive care unit patients diagnosed with AKI to assess the effects of post-admission amoxicillin administration on 30-day and 90-day mortality rates and acute kidney disease incidence. We employed Cox proportional hazards models, propensity score matching, and inverse probability of treatment weighting to control for potential confounders.

**Results:**

Among 24,650 AKI patients, 676 (2.7%) received amoxicillin. The results indicated significantly lower mortality rates at 30 days (hazard ratio [HR] 0.54, 95% confidence interval [CI] 0.42–0.69) and 90 days (HR 0.64, 95% CI 0.52–0.77) in the amoxicillin group compared to non-recipients. Additionally, amoxicillin administration was associated with a reduced incidence of AKD (HR 0.49, 95% CI 0.36–0.65) but resulted in a modestly increased length of hospital stay (mean difference [MD] 1.95 days, 95% CI 1.15–2.75). A dose‒response relationship was evident, with higher doses (>875 mg) further decreasing mortality rates. Subgroup analysis revealed consistent benefits across most patient groups.

**Conclusion:**

Amoxicillin administration following ICU admission in patients with AKI was associated with improved survival rates and a lower incidence of AKD, highlighting its potential as a therapeutic measure for AKI management.

## 1 Introduction

Acute kidney injury (AKI) represents a significant medical challenge, affecting approximately one-third of patients in intensive care units (ICUs) (Mehta et al., 2015). This condition is associated with increased mortality rates both in the short and long term and elevates the risk of progressing to chronic kidney disease (CKD), particularly in severe cases of AKI (Levey and James, 2017; Hoste et al., 2018). Despite advancements in critical care and dialysis technologies, the development of effective therapeutic strategies for AKI remains limited (Pickkers et al., 2021).

The gut microbiota has emerged as a pivotal factor in the pathophysiology of AKI, mediated through intricate interactions between the host immune system and the ecosystem of the gastrointestinal tract (Andrade-Oliveira et al., 2015; Nakade et al., 2018; Yang et al., 2020). Recently, a groundbreaking study by Gharaie et al. revealed that the administration of antibiotics, specifically amoxicillin, can facilitate the repair process in ischemic AKI by modulating the gut microbiota, even when administered after injury (Gharaie et al., 2023). Additionally, a study conducted by Jeonghwan et al. suggested that amoxicillin-induced intestinal microbiota depletion can attenuate the AKI-to-CKD transition via NADPH oxidase 2 and trimethylamine-N-oxide inhibition (Lee et al., 2024).

Amoxicillin, a widely prescribed penicillin and a beta-lactam antibiotic, has traditionally been associated with adverse effects on renal function, with high intravenous doses implicated in the onset of AKI (Garnier et al., 2020; Mousseaux et al., 2021). This perspective, however, is being challenged by emerging evidence that underscores the protective effect of amoxicillin in acute ischemic scenarios. Nakamura et al. demonstrated that a 10-day pretreatment with amoxicillin effectively prevented acute ischemic liver injury in a mouse model (Nakamura et al., 2019). Furthermore, recent studies by Gharaie et al. and Jeonghwan et al. suggested that amoxicillin following severe AKI could represent a promising novel therapeutic strategy to accelerate the recovery of kidney function (Gharaie et al., 2023; Lee et al., 2024). Despite these findings, the effectiveness of amoxicillin in clinical settings has not been well-established, and fundamental studies on treating AKI with amoxicillin are still limited.

We therefore aimed to investigate the impact of amoxicillin administration on the prognosis of AKI in ICU patients. We focused on evaluating whether amoxicillin exposure following ICU admission correlates with a decrease in mortality rates at 30 and 90 days. Furthermore, we explored the association between amoxicillin treatment and a reduced incidence of acute kidney disease, characterized by AKI lasting beyond 7 days, up to 90 days.

## 2 Method

### 2.1 Data sources

The Medical Information Mart for Intensive Care IV ver 2.0 (MIMIC-IV, version 2.0) is a large and openly accessible critical care database. It includes more than 70,000 ICU admissions hospitalized at the Beth Israel Deaconess Medical Center (Boston, United States) from 2008 to 2019. Authorization to access and utilize this database was granted to one of the authors after successfully completing the Protecting Human Research Participants course offered by the National Institutes of Health (LX, certification number 12059504).

Permission for the establishment of this database was obtained from the Institutional Review Boards of both the Massachusetts Institute of Technology (MIT, Cambridge, MA, United States) and the Beth Israel Deaconess Medical Center. This project complied with the Helsinki Declaration, and no additional ethics approval was necessary. To ensure privacy, all patient data included were anonymized. This retrospective observational study adhered to the guidelines of the Strengthening the Reporting of Observational Studies in Epidemiology (STROBE) initiative.

### 2.2 Population selection criteria

Eligible patients with AKI were those who were older than 18 years old at admission and who had been hospitalized for more than 48 h. Patients were excluded from our study if 1) >5% of their individual data were missing or 2) the baseline values exceeded the median ±1.5 times the interquartile range. AKI diagnosis followed the Kidney Disease: Improving Global Outcomes (KDIGO) guidelines (Lameire et al., 2021), which specify changes in serum creatinine (SCr) levels and urine output. Stage 1 is defined as an increase ≥1.5 times the baseline SCr within the prior 7 days or 0.3 mg/dL in the SCr within 48 h or a urine output <0.5 mL/kg/h per 6 h. Stage 2 is characterized by an increase in SCr ≥2.0 times the baseline or a urine output <0.5 mL/kg/h per 12 h. Stage 3 is characterized by an increase in SCr ≥3.0 times baseline, SCr ≥4.0 mg/dL, initiation of renal replacement therapy (RRT), or urine output <0.3 mL/kg/h per 24 h. Patients who were diagnosed with AKI 6 h before to 48 h after ICU admission were included in the study. Baseline SCr was identified as the lowest value within 7 days or 48 h prior to AKI diagnosis.

### 2.3 Data extraction

Patient data were retrieved from the MIMIC-IV database using Structured Query Language (SQL) via PostgreSQL tools (version 15.1). The dataset included patient identifiers, clinical and laboratory parameters, comorbidities, and scoring systems. The clinical parameters included age, sex, ethnicity, heart rate, systolic blood pressure (SBP), percutaneous oxygen saturation (SPO2), diastolic blood pressure (DBP), vasopressin use, ventilator use, and RRT. The following laboratory parameters were measured: anion gap, blood urea nitrogen (BUN) level, SCr level, potassium level, red blood cell (RBC) count, red cell distribution width (RDW), and international normalized ratio (INR).

The scores, including the Sequential Organ Failure Assessment (SOFA) score and Glasgow Coma Scale (GCS) score, were calculated for each patient. Only the data from the first admission to the ICU were collected for patients who had multiple admissions to the ICU. The following comorbidities were identified: hypertension, diabetes, coronary artery disease, chronic obstructive pulmonary disease (COPD), malignancy, hematologic disease, atrial fibrillation, liver disease, shock, and sepsis based on the ninth or 10th revision of the International Classification of Diseases (ICD-9/10) code. The first measured values within 6 h before ICU admission and within 48 h after ICU admission were used as the baseline data. Amoxicillin exposure was defined as the administration of at least one dose of amoxicillin orally or via a nasogastric tube between 48 h before ICU admission and ICU discharge.

### 2.4 Outcomes

The primary outcome of our study focused on 30-day all-cause mortality. The secondary outcomes included 90-day mortality, incidence of AKD, duration of ICU stay, and total length of hospital stay. AKD diagnosis followed the 2017 Acute Disease Quality Initiative-16 (ADQI-16) criteria, characterizing AKD as AKI persisting for more than 7 days following the initial AKI event.

### 2.5 Statistical analysis

Baseline characteristics were categorized based on whether patients were treated with amoxicillin and are presented as frequencies (percentages) for categorical variables and means (standard deviations) or medians (interquartile ranges) for continuous variables. For group comparisons, the chi-square test was used for categorical data. For continuous variables, we employed analysis of variance or the Mann–Whitney *U* test as appropriate. We accounted for missing baseline data by using multiple imputation.

We used a stepwise selection method to choose variables for inclusion in the Cox proportional hazards models. This method involves iteratively adding or removing variables based on statistical criteria to identify the most significant predictors. Cox proportional hazards models were constructed to test the associations between amoxicillin administration and outcomes, with adjustment for all potential confounding variables, including age, sex, ethnicity, vasoactive use, RRT, GCS score, SOFA score, RDW, anion gap, potassium, BUN, SCr, INR, heart rate, SPO2, shock, sepsis, coronary artery disease, liver disease, atrial fibrillation, and COPD (listed in Table 1).

**TABLE 1:** Baseline characteristics of the study population.

Characteristic	Use of amoxicillin			*p*-value
	All patients (n = 24,650)	Non-amoxicillin (n = 23,974)	Amoxicillin (n = 676)	
Age, years	67.92 (15.53)	67.93 (15.49)	67.79 (17.16)	0.82
Sex, n (%)				0.977
Female	10,351 (42.0)	10,068 (42.0)	283 (41.9)	
Male	14,299 (58.0)	13,906 (58.0)	393 (58.1)	
Ethnicity, n (%)				0.812
Black	2059 (8.4)	1998 (8.3)	61 (9.0)	
Other	5694 (23.1)	5540 (23.1)	154 (22.8)	
White	16,897 (68.5)	16,436 (68.6)	461 (68.2)	
SBP, mmHg	123.06 (24.98)	122.99 (25.00)	125.51 (24.44)	0.01
DBP, mmHg	67.06 (17.97)	67.00 (17.97)	68.93 (17.77)	0.006
Heart rate, beats/minute	85.00 [75.00, 100.00]	85.00 [75.00, 100.00]	87.00 [75.00, 102.00]	0.083
SpO2, %	99.00 [96.00, 100.00]	99.00 [96.00, 100.00]	98.00 [95.75, 100.00]	0.038
Hypertension, n (%)	14,370 (58.3)	14,000 (58.4)	370 (54.7)	0.062
Diabetes, n (%)	7999 (32.5)	7783 (32.5)	216 (32.0)	0.811
Coronary artery disease, n (%)	9320 (37.8)	9114 (38.0)	206 (30.5)	<0.001
COPD, n (%)	2788 (11.3)	2706 (11.3)	82 (12.1)	0.535
Liver disease, n (%)	3534 (14.3)	3436 (14.3)	98 (14.5)	0.948
Atrial fibrillation (%)	4094 (16.6)	3978 (16.6)	116 (17.2)	0.735
Shock (%)	3296 (13.4)	3212 (13.4)	84 (12.4)	0.5
Sepsis (%)	14,560 (59.1)	14,096 (58.8)	464 (68.6)	<0.001
Anion gap, mmol/L	15.00 [12.00, 18.00]	15.00 [12.00, 18.00]	15.00 [13.00, 18.00]	<0.001
BUN, mg/dL	20.00 [14.00, 32.00]	20.00 [14.00, 32.00]	21.00 [15.00, 33.00]	0.134
Serum creatinine, mg/dL	1.00 [0.80, 1.50]	1.00 [0.80, 1.50]	1.10 [0.80, 1.60]	0.27
Serum potassium, mmol/L	4.20 [3.80, 4.70]	4.20 [3.80, 4.70]	4.20 [3.80, 4.70]	0.547
INR	1.30 [1.10, 1.50]	1.30 [1.10, 1.50]	1.30 [1.10, 1.50]	0.653
RBC, 10^12^/L	3.60 [3.02, 4.21]	3.59 [3.02, 4.21]	3.65 [3.03, 4.20]	0.483
RDW, %	14.30 [13.30, 15.80]	14.30 [13.30, 15.80]	14.50 [13.40, 16.00]	0.013
SOFA	5.00 [3.00, 8.00]	5.00 [3.00, 8.00]	5.00 [3.00, 7.00]	0.005
GCS	14.00 [10.00, 15.00]	14.00 [10.00, 15.00]	14.00 [11.75, 15.00]	0.135
AKI KDIGO stage, n (%)				0.558
Stage 1	7344 (29.8)	7155 (29.8)	189 (28.0)	
Stage 2	12,441 (50.5)	12,093 (50.4)	348 (51.5)	
Stage 3	4865 (19.7)	4726 (19.7)	139 (20.6)	
Renal replacement therapy, n (%)	1287 (5.2)	1264 (5.3)	23 (3.4)	0.039
Vasoactive use (%)	11,753 (47.7)	11,508 (48.0)	245 (36.2)	<0.001
Ventilator use (%)	12,436 (50.5)	12,127 (50.6)	309 (45.7)	0.014

Abbreviations: SBP, systolic blood pressure; DBP, diastolic blood pressure, SpO2 percutaneous oxygen saturation, COPD, chronic obstructive pulmonary disease; BUN, blood urea nitrogen; INR, international normalized ratio; RBC, red blood count; RDW, red cell distribution width; SOFA, sequential organ failure assessment; GCS, glasgow coma scale; KDIGO, Kidney Disease: Improving Global Outcomes. Normally distributed data are presented as the mean (SD) (analysis of variance); non-normally distributed data are presented as median (IQR) (nonparametric Wilcoxon test); and categorical variables are presented as n (%) (chi-square test).

We conducted a series of sensitivity analyses to rigorously test the robustness of our outcomes. Initially, a preplanned multivariable Cox regression analysis was performed. Subsequently, to address potential confounding factors, we employed propensity score matching (PSM), aligning patients based on whether they received amoxicillin. A caliper width of 0.2 was used to ensure close matching of patient characteristics. The balance between the groups was evaluated using the standard mean difference (SMD), with a threshold SMD of 0.1 set as the criterion for significant baseline imbalance (Austin, 2011). After matching, we analyzed the outcomes using a Cox regression model to confirm the consistency of our findings.

Furthermore, to further refine our adjustment for potential confounders, we performed inverse probability of treatment weighting (IPTW) analysis. Weights for this analysis were derived from a multivariable Cox regression model incorporating the same covariates as the primary analysis. This method assigns weights to each patient based on the inverse probability of receiving the treatment they actually received, thus creating a synthetic sample in which the distribution of measured covariates is independent of treatment assignment (Raad et al., 2020; Chesnaye et al., 2022). The impact of amoxicillin on the risk of 30-day and 90-day mortality, as well as AKD, was then estimated by weighted Cox regression model with IPTW.

For outcomes including ICU length of stay and hospital length of stay, we employed linear regression models, adjusting for the covariates outlined earlier in our analysis.

Potential effect modifications by sex, ethnicity, age, GCS score, SOFA score, shock, sepsis, vasoactive use, and AKI stage were assessed by subgroup and interaction analyses for the primary outcome. Subgroup analyses were performed using multivariable Cox regression models, with a *p*-value for interactions less than 0.1 indicating a significant interaction.

Outcomes were measured as hazard ratios (HRs) for categorical variables and mean differences (MDs) for continuous variables, along with their 95% confidence intervals (CIs). Data analysis was conducted using R software version 4.2.2. A two-tailed *p*-value less than 0.05 was considered to indicate statistical significance.

## 3 Results

### 3.1 Subject characteristics

**FIGURE 1:**
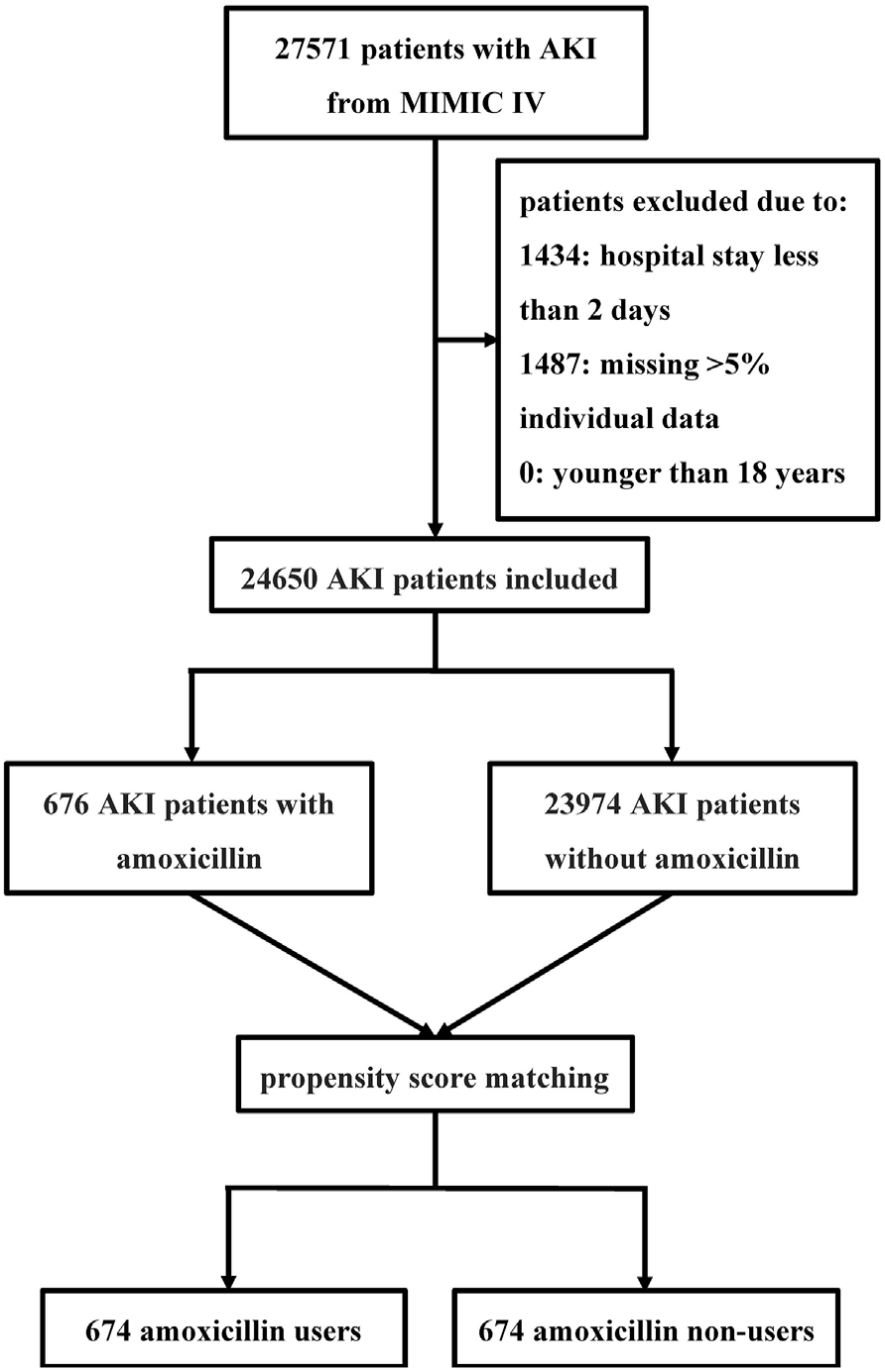
Flowchart of the study Abbreviations: MIMIC IV Multiparameter Intelligent Monitoring in Intensive Care Database IV; AKI acute kidney injury.

A total of 27,571 critically ill patients with AKI were included in our study. After excluding patients according to the exclusion criteria, 24,650 patients with AKI were included in the analysis (Figure 1). Among these, 676 (2.7%) patients had been exposed to amoxicillin, while 23,974 (97.3%) had not received the antibiotic. The average age of the study participants was 67.9 years, with males comprising 58.0% (14,299) and females comprising 42.0% (10,351) of the sample.

Patient characteristics are detailed in Table 1. Notably, individuals in the non-amoxicillin group were more likely to present with sepsis and malignancy but less likely to suffer from coronary artery disease. Additionally, this group exhibited higher SOFA scores and lower levels of anion gap and RDW than did the amoxicillin group. Moreover, there were no significant differences in GCS score, BUN level, SCr level, or AKI stage between the two groups.

### 3.2 Associations between amoxicillin and 30-day mortality and 90-day mortality

**FIGURE 2:**
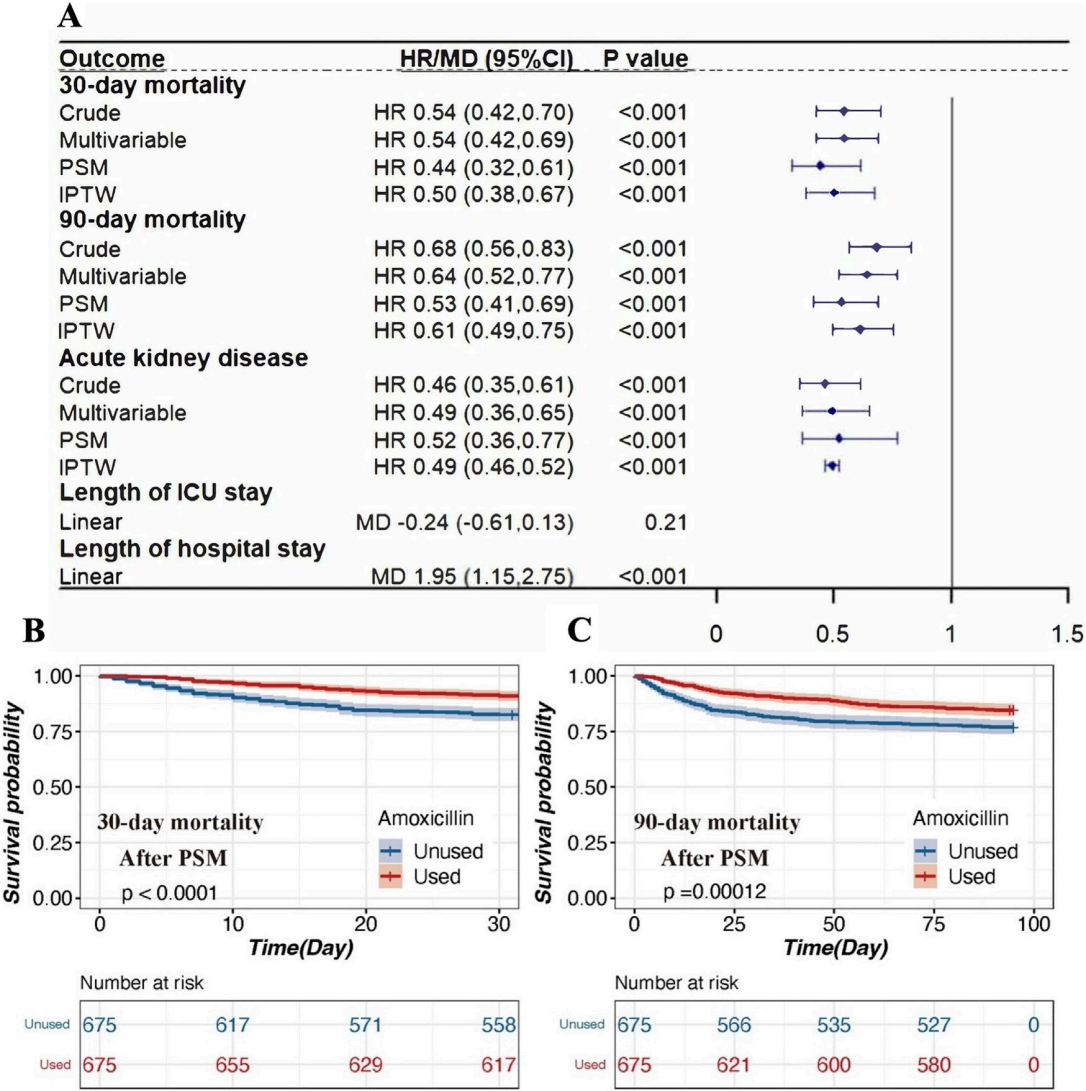
The association between amoxicillin administration and clinical outcomes in patients with AKI. **(A)**. Association between amoxicillin administration and clinical outcomes. Four different methods were used to address the associations: 1) univariable Cox regression, 2) multivariable Cox regression, 3) propensity score matching, and 4) inverse propensity weighted modeling. **(B)**. Kaplan‒Meier survival curves of the amoxicillin group and non‐amoxicillin group after PSM for 30‐day mortality. **(C)**. Kaplan‒Meier survival curves of the amoxicillin group and non-amoxicillin group after PSM for 30‐day mortality. Notes: HRs (95% CIs) were derived from Cox proportional hazards regression models. Covariates were adjusted as in the model II. The MDs (95% CIs) were derived from linear regression models. Covariates were adjusted as in the model II. Abbreviations: HR hazard ratio; MD mean difference; PSM propensity score matching; IPTW inverse probability of treatment weighting; ICU intensive care unit.

The 30-day mortality rate was significantly lower in the amoxicillin group (9.0%) compared to the non-amoxicillin group (15.8%) with a hazard ratio (HR) of 0.54 (95% confidence interval [CI] 0.42–0.70, *p* < 0.001) (Figure 2). Similarly, the 90-day mortality rate was also lower in the amoxicillin group (15.4%) compared to the non-amoxicillin group (21.2%), with an HR of 0.68 (95% CI 0.56–0.83, *p* < 0.001) (Figure 2).

Even after adjusting for clinically relevant covariates, the association between amoxicillin administration and reduced mortality remained significant. The adjusted HR for 30-day mortality was 0.54 (95% CI 0.42–0.69, *p* < 0.001), and for 90-day mortality, it was 0.64 (95% CI 0.52–0.77, *p* < 0.001) (Figure 2).

After PSM, 674 patients who received amoxicillin were matched with 674 patients who did not (Supplemental Table S1). This analysis demonstrated that amoxicillin administration was associated with significantly lower 30-day mortality (HR = 0.44, 95% CI 0.32–0.61, *p* < 0.001) and 90-day mortality (HR = 0.53, 95% CI 0.41–0.69, *p* < 0.001) (Figure 2). The IPTW analyses also supported these findings, showing that the amoxicillin group had lower 30-day mortality (HR = 0.50, 95% CI 0.38–0.67, *p* < 0.001) and 90-day mortality (HR = 0.61, 95% CI 0.49–0.75, *p* < 0.001) compared to the non-amoxicillin group (Figure 2).

**FIGURE 3:**
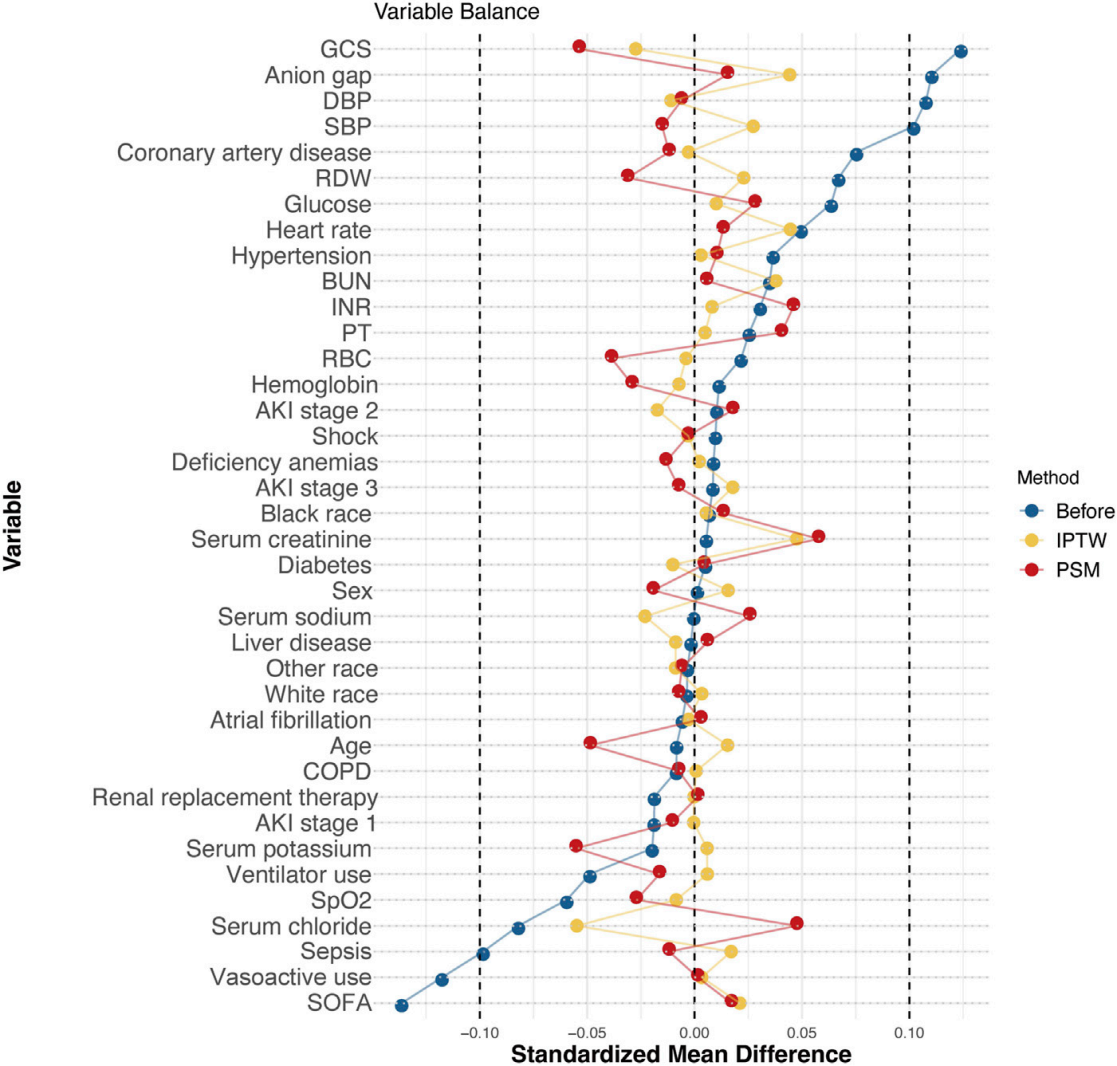
Love plot of balance in baseline characteristics before and after PSM and IPTW in the MIMIC-IV database. Note: Balance in baseline characteristics before and after PSM and IPTW in the MIMIC-IV database. After PSM and IPTW, the standardized mean differences in the baseline and clinical characteristics were well balanced. Abbreviations: GCS, Glasgow Coma Scale; DBP, diastolic blood pressure; SBP, systolic blood pressure; RDW, red cell distribution width; BUN, blood urea nitrogen; INR, international normalized ratio; PT, prothrombin time; RBC, red blood cell; COPD, chronic obstructive pulmonary disease; SpO2, percutaneous oxygen saturation; SOFA, Sequential Organ Failure Assessment.

Figure 3 shows the SMDs for all the individual covariates. After PSM and IPTW, the baseline profiles were all balanced between the two groups, with an SMD<10% for all variables.

According to the Kaplan‒Meier survival curve for 30-day mortality, mortality was lower for the amoxicillin group than for the non-amoxicillin group (HR = 0.54; *p* < 0.001). After PSM, the Kaplan-Meier survival curve for 30-day mortality remained lower for the amoxicillin group than for the non-amoxicillin group (HR = 0.44; *p* < 0.001), as noted in Figure 2. Similar results were observed for 90-day mortality.

### 3.3 Associations between amoxicillin and AKD incidence and length of stay

The incidence of AKD was lower in patients exposed to amoxicillin compared to those who were not. The unadjusted HR was 0.46 (95% CI 0.35–0.61, *p* < 0.001). After adjusting for relevant covariates, the HR was 0.49 (95% CI 0.36–0.65, *p* < 0.001). Post-PSM analysis yielded an HR of 0.52 (95% CI 0.36–0.77, *p* < 0.001), and the IPTW analysis showed an HR of 0.49 (95% CI 0.46–0.52, *p* < 0.001) (Figure 2). Nevertheless, amoxicillin use was associated with a longer length of hospital stay (MD = 1.95, 95% CI 1.15–2.75, *p* < 0.001) (Figure 2).

### 3.4 Association between amoxicillin dose and mortality

**FIGURE 4:**
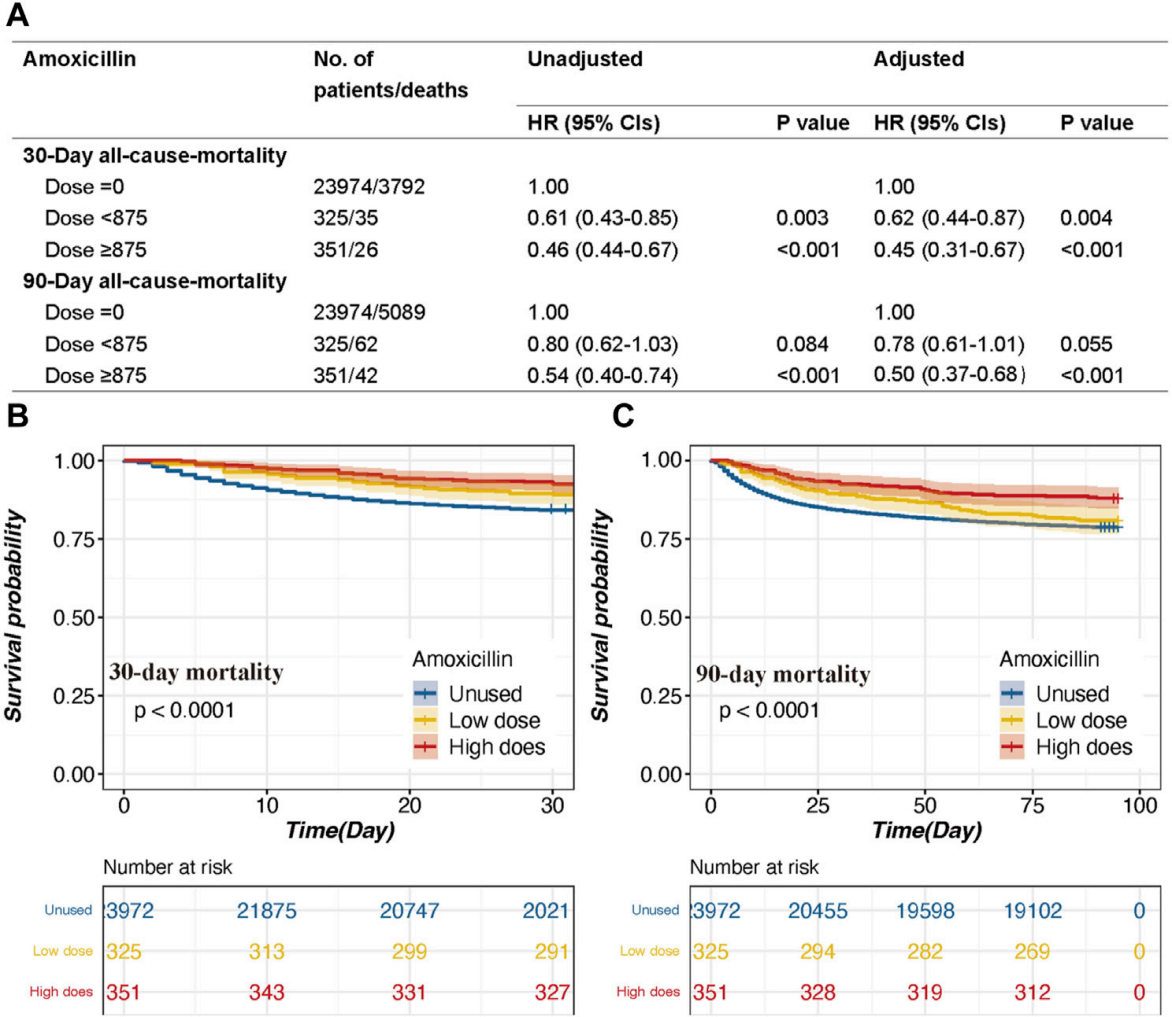
The association between survival outcomes and different dosages and the Kaplan‒Meier survival curves of the high-dose group, low-dose group and non-amoxicillin group. **(A)**. HRs (95% CIs) for all-cause mortality across groups divided by therapeutic dose. **(B)**. Group for 30-day mortality. **(C)**. 90-day mortality group. Models I and II were derived from Cox proportional hazards regression models; Model I covariates were adjusted for age, ethnicity, and sex; Model II covariates were adjusted for age, ethnicity, vasoactive use, renal replacement therapy, GCS score, SOFA score, RDW, potassium, chloride, anion gap, MCV, BUN, serum creatinine, urine output, INR, white blood cell count, heart rate, temperature, SpO2, malignancy, shock, deficiency anemia, sepsis, coronary artery disease, liver disease, atrial fibrillation, and chronic obstructive pulmonary disease.

The amoxicillin group can be divided into two groups based on the dose of amoxicillin: the high-dose amoxicillin group (dose ≥875 mg), which consisted of 1.4% (n = 351) of patients, and the low-dose amoxicillin group (dose <875 mg), which consisted of 1.3% (n = 325) of patients. In multivariate analysis, the amoxicillin dose was analyzed to determine whether it was independently associated with all-cause mortality (Figure 4).

In model I adjusted for age, ethnicity, and sex, the high-dose group was associated with a decreased risk of 30-day mortality (*p* < 0.001) and 90-day mortality (*p* < 0.001) (Figure 4). The HRs (95% CIs) for the high-dose group were 0.46 (0.44–0.67) and 0.54 (0.40–0.74), respectively (Figure 4). Moreover, the low-dose group had a decreased risk of 30-day mortality (*p* = 0.003) (Figure 4). The HR (95%CI) for the low-dose group was 0.61 (0.43–0.85) (Figure 4).

In model II, after adjusting for the aforementioned covariates, a high dose of amoxicillin remained a significant predictor of all-cause mortality at 30 days and 90 days (HR, 95% CI: 0.45, 0.31–0.67; 0.50, 0.37–0.68, both *p* < 0.001), while a low dose of amoxicillin was also associated with decreased 30-day mortality (HR, 95% CI: 0.62, 0.44–0.87) (Figure 4). The Kaplan‒Meier survival curve also indicated that the high-dose group had lower mortality than the low-dose group (Figure 4).

### 3.5 Subgroup analysis

**FIGURE 5:**
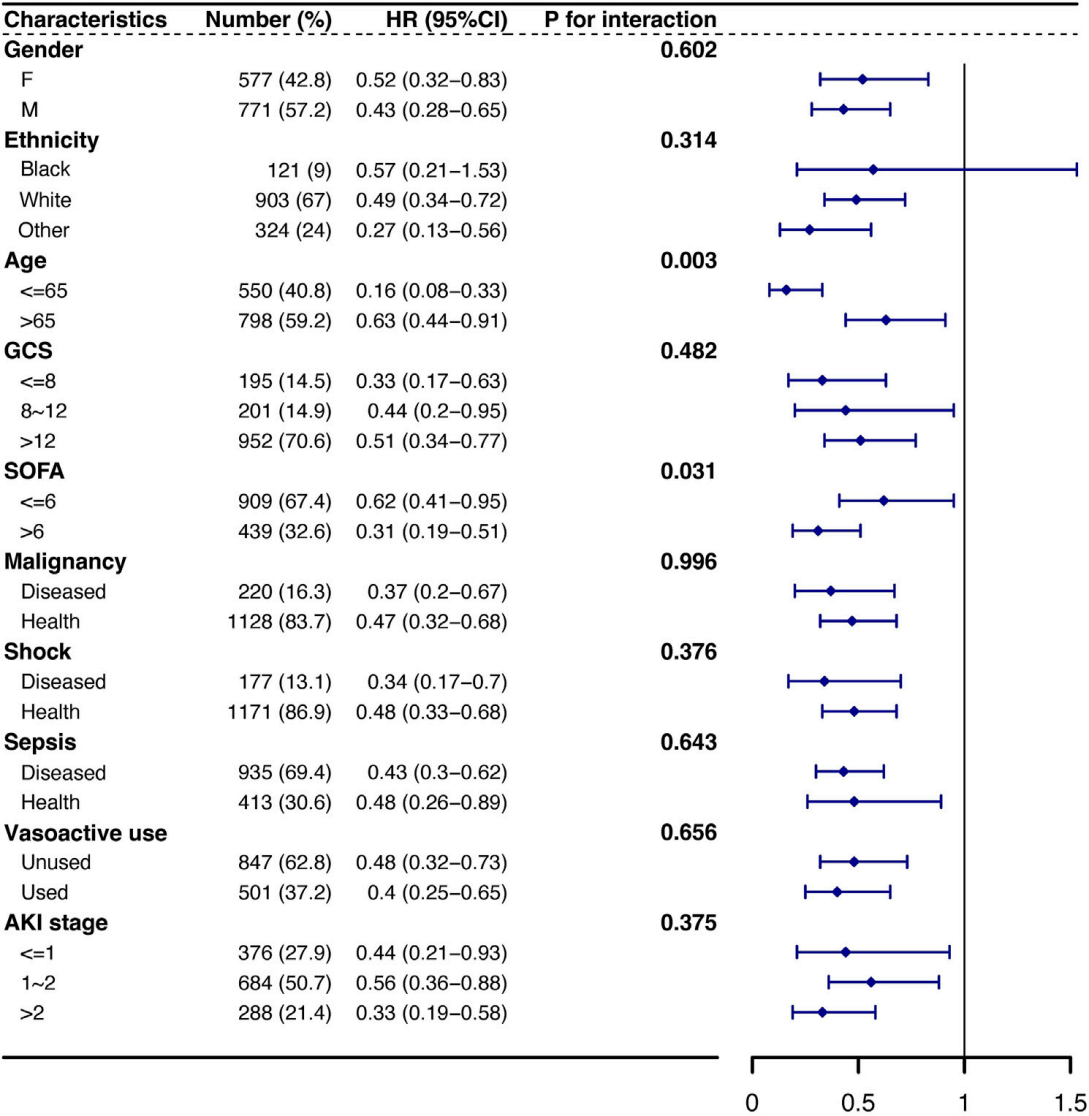
Subgroup analysis of the association between 30-day mortality and amoxicillin administration. Note: HRs (95% CIs) were derived from Cox proportional hazards regression models. Covariates were adjusted as in the model II. Abbreviations: HR hazard ratio, GCS Glasgow Coma Scale, SOFA Sequential Organ Failure Assessment, AKI acute kidney injury.

According to the subgroup analysis, the association between amoxicillin and the risk of 30-day mortality was similar in most strata (Figure 5). Although significant interactions were observed for subgroups stratified by age and SOFA score, the direction of the association between amoxicillin and the risk of 30-day mortality remained stable in most strata, except for ethnicity. For patients of white and other ethnicities, the use of amoxicillin was associated with decreased mortality (HR, 95% CI: 0.49, 0.34–0.72; HR, 95% CI: 0.27, 0.13–0.56), while no significant difference in mortality existed for black patients (HR, 95% CI: 0.57, 0.21–1.53) (Figure 5).

## 4 Discussion

Our study, analyzing the population with AKI, revealed that amoxicillin administration is correlated with reduced 30-day mortality and 90-day mortality and decreased AKD incidence. Notably, a dose-dependent attenuation in mortality was observed with amoxicillin dosages between 875 mg and 2000 mg. To test the association between amoxicillin administration and patient outcomes, we constructed Cox proportional hazards models with adjusted potential confounding variables. Kaplan‒Meier survival curves also demonstrated this pattern. Furthermore, we employed PSM and IPTW analysis to further refine our adjustment for potential confounding variables. Subgroup analyses were performed to show significant interactions.

β-Lactam antibiotics, which are crucial for treating bacterial infections, have been implicated in increasing AKI risk, particularly when combined with vancomycin (Downes et al., 2017; Lima et al., 2020). Previous research suggested that high β-lactam dosages are a potential cause of AKI, particularly at doses above 8 g in total (Bellos et al., 2020). Our research diverges by focusing on lower amoxicillin dosages, where we observed a significant decrease in mortality, particularly at doses above 875 mg and below 2000 mg.

In our study, we focused on individuals suffering from AKI and discovered that amoxicillin was associated with decreased 30-day mortality and 90-day mortality. In addition to reducing mortality, we observed a notable decrease in the incidence of AKD, further confirming the efficacy of amoxicillin in improving AKI prognosis and renal function, which may slow the progression of AKI to CKD. Recent investigations have proposed that the pharmacological effects of amoxicillin may be intricately linked to alterations within the intestinal microbiome (Palleja et al., 2018; Zimmermann and Curtis, 2019). The gut microbiota, which is essential for gastrointestinal homeostasis, is widely recognized to significantly impact renal function (Dalile et al., 2019; Gharaie et al., 2020; Chou et al., 2022; Portincasa et al., 2022). A recent study by Gharaie et al. utilizing a murine model demonstrated that amoxicillin can expedite renal recovery following severe AKI, as evidenced by improved glomerular filtration rates and diminished fibrosis (Gharaie et al., 2023). This regenerative process is facilitated through modifications in renal CD8^+^ T cells and PD1+CD8^+^ cells. Additionally, Jeonghwan et al. revealed that antibiotic-induced intestinal microbiota depletion can attenuate the AKI-to-CKD transition. This study indicated that plasma trimethylamine N-oxide (TMAO) is a key metabolite associated with the AKI-to-CKD transition and that NADPH oxidase 2 (NOX2) activation is a key regulator of the TMAO-related AKI-to-CKD transition. Antibiotic-induced microbiota depletion successfully inhibited gut microbiome-derived TMAO metabolites and attenuated inflammation, apoptosis, G2/M arrest, and fibrosis via NOX2 suppression (Lee et al., 2024). Furthermore, the influence of antibiotics on the gut microbiota in germ-free mice substantiated their contributory role in renal repair (Gong et al., 2019). Our findings align with these findings, suggesting that the administration of amoxicillin may be a beneficial approach for improving AKI prognosis.

Our results demonstrated that amoxicillin use was associated with a longer length of hospital stay, which may reflect that those who did not receive amoxicillin potentially have higher mortality. Thus, an extended length of hospital stay might indicate lower mortality and better treatment outcomes, which is consistent with our findings. The subgroup analyses showed that the association between amoxicillin and the risk of 30-day mortality was similar in most strata. Regarding demographic variations, our results showed no significant difference in the effect of amoxicillin on black patients. However, the small sample size (9%) of black patients and the potential impact of gut microbiota diversity across ethnicities warrant further investigation (Syromyatnikov et al., 2022).

A consensus report of the Acute Disease Quality Initiative 16 Workgroup suggested that treatment for infection with an antibiotic for survival is necessary and might prevent or ameliorate AKI by treating underlying causes and complications. On the other hand, combining nephrotoxins can result in pharmacodynamic drug interactions, which may lead to the worsening of the disease (Chawla et al., 2017). A prospective study is required to clarify the appropriate dose of amoxicillin for the treatment of AKI in the future.

There are several limitations to our study. First, most of the patients we included did not receive amoxicillin, so after PSM, the sample size was relatively small. Second, we could not evaluate the upper limit of the proper dosage of amoxicillin. To determine the therapeutic effect of amoxicillin, additional clinical studies and randomized controlled trials should be performed. Third, despite the multivariable analysis and propensity score matching, residual confounding factors cannot be fully excluded. Fourth, due to the lack of follow-up serum creatinine data after ICU discharge, we were unable to assess the long-term renal recovery of the patients. Finally, this study was conducted using data from a single center. Therefore, these findings should be validated in multicenter trials to ensure their generalizability across different populations and clinical settings.

## 5 Conclusion

The administration of amoxicillin is associated with lower 30-day mortality and 90-day mortality and a decreased incidence of AKD in AKI patients in the ICU. These outcomes suggest that amoxicillin might be a therapeutic intervention for a better AKI prognosis. Additional studies are needed to determine the efficiency, dosage, and duration of amoxicillin for AKI treatment.

## Data Availability

The original data presented in the study are included in the article and its Supplementary Material. Further inquiries can be directed to the corresponding authors.
