# Corrigendum: Association between amoxicillin administration and outcomes in critically ill patients with acute kidney injury

**DOI:** 10.3389/fphar.2024.1468612

**Published:** 2024-11-05

**Authors:** Xinyao Luo, Weijian Zhou, Dingyuan Wan, Jing Peng, Ruoxi Liao, Baihai Su

**Affiliations:** ^1^ Department of Nephrology, Kidney Research Institute, West China Hospital, Sichuan University, Chengdu, China; ^2^ Department of Intensive Care Medicine, West China Hospital, Sichuan University, Chengdu, China

**Keywords:** acute kidney injury, amoxicillin, intensive care unit, mortality, acute kidney disease

In the published article, there was an error in Figure 2 as published. Figure 2 contains superfluous elements. The corrected Figure 2 and its caption appear below.

**FIGURE 2:**
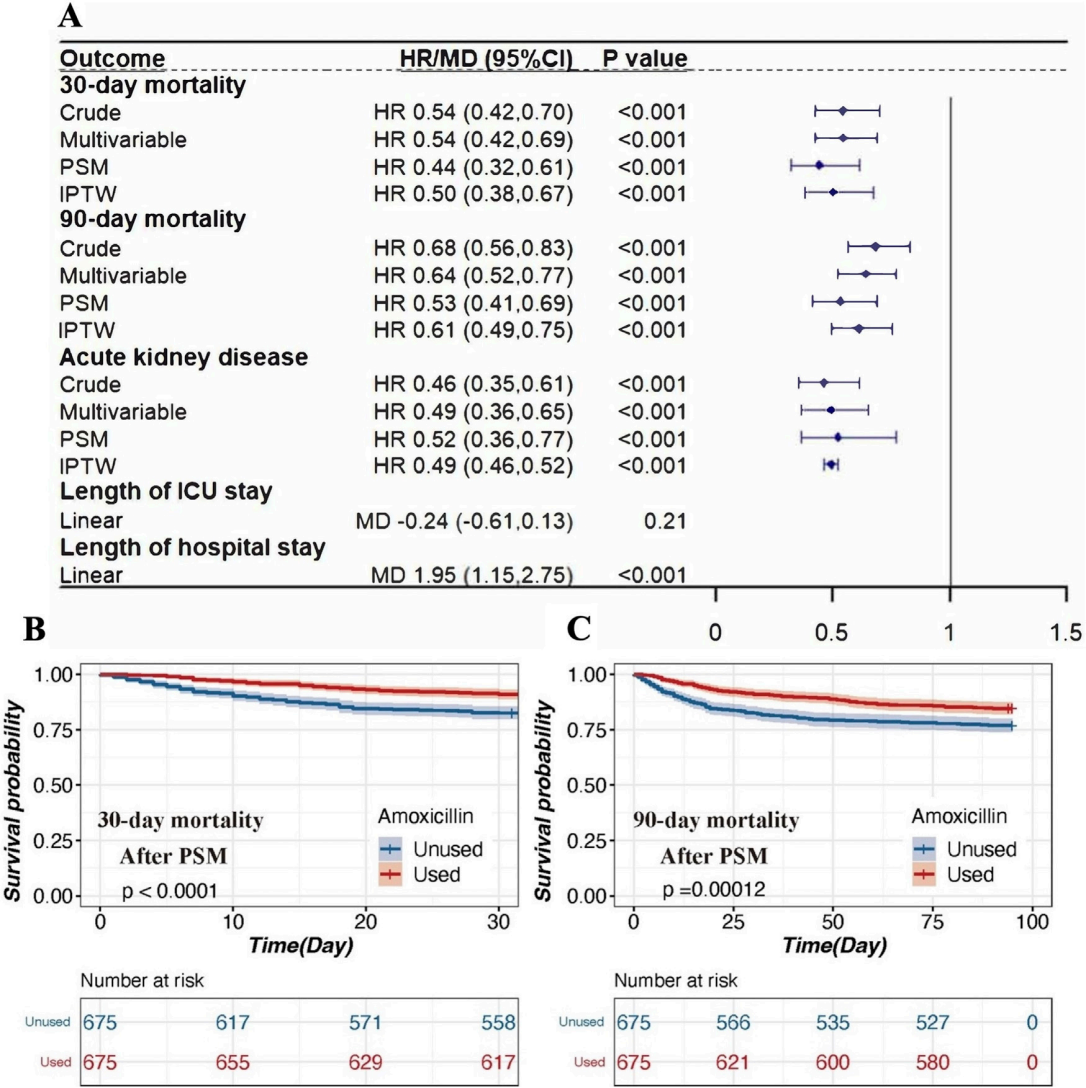
The association between amoxicillin administration and clinical outcomes in patients with AKI. **(A)** Association between amoxicillin administration and clinical outcomes. Four different methods were used to address the associations: 1) univariable Cox regression, 2) multivariable Cox regression, 3) propensity score matching, and 4) inverse propensity weighted modeling. **(B)** Kaplan‒Meier survival curves of the amoxicillin group and non-amoxicillin group after PSM for 30-day mortality. **(C)** Kaplan‒Meier survival curves of the amoxicillin group and non-amoxicillin group after PSM for 30-day mortality. Notes: HRs (95% CIs) were derived from Cox proportional hazards regression models. Covariates were adjusted as in the model II. The MDs (95% CIs) were derived from linear regression models. Covariates were adjusted as in the model II. Abbreviations: HR, hazard ratio; MD mean difference; PSM, propensity score matching; IPTW, inverse probability of treatment weighting; ICU, intensive care unit.

The authors apologize for this error and state that this does not change the scientific conclusions of the article in any way. The original article has been updated.

