# Supplementary material for: Association between amoxicillin administration and outcomes in critically ill patients with acute kidney injury
Source: Front Pharmacol. 2024 Jul 15;15:1409654. doi: 10.3389/fphar.2024.1409654 (PMC11284156; doi:10.3389/fphar.2024.1409654)
Supplement: Supplementary file 1 [file DataSheet1.PDF]

Supplemental Figure 1

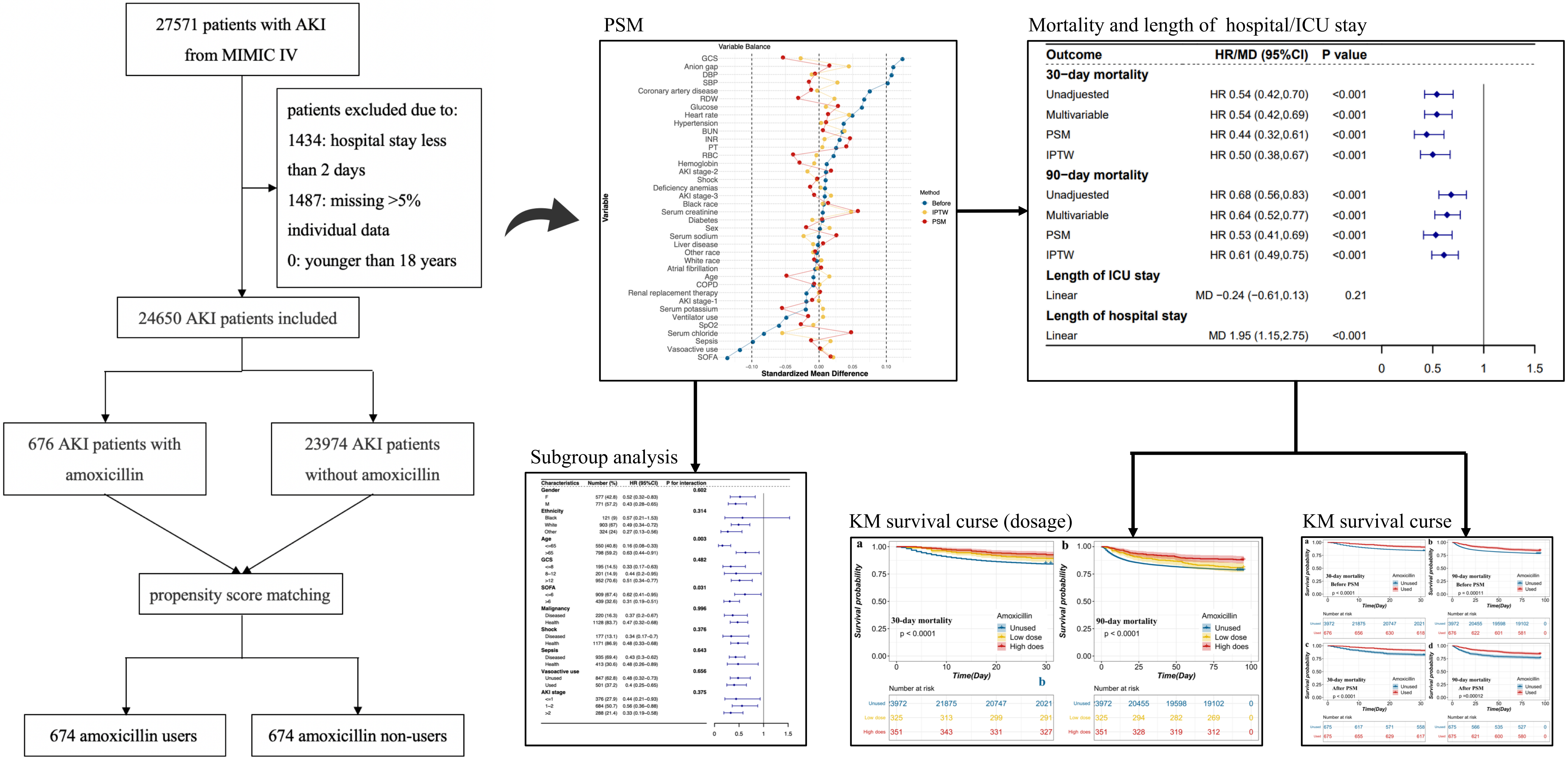

Supplemental Figure 1. Study design of the clinical investigation.

Abbreviations: MIMIC IV Multiparameter Intelligent Monitoring in Intensive Care Database IV, AKI acute kidney injury, PSM propensity score matching, KM Kaplan-Meier.

Supplemental Table 1. Baseline characteristics of the study population after propensity score matching

Characteristic	use of amoxicillin （matched data）			P value	SMD
	All patients (n=1348)	non-amoxicillin (n=674)	amoxicillin (n=674)		
Age, years	67.71 (16.58)	67.62 (15.96)	67.79 (17.18)	0.853	0.01
Sex, n (%)				0.509	0.039
Female	577 (42.8)	295 (43.8)	282 (41.8)		
Male	771 (57.2)	379 (56.2)	392 (58.2)		
Ethnicity, n (%)				0.515	0.063
Black	121 (9.0)	60 (8.9)	61 (9.1)		
Other	324 (24.0)	171 (25.4)	153 (22.7)		
White	903 (67.0)	443 (65.7)	460 (68.2)		
SBP, mmHg	125.20 (24.17)	124.84 (23.88)	125.56 (24.46)	0.584	0.03
DBP, mmHg	68.47 (17.61)	67.97 (17.44)	68.97 (17.78)	0.297	0.057
Heart rate, beats/minute	86.00 [74.00, 100.00]	85.00 [74.00, 100.00]	87.00 [75.00, 102.00]	0.162	0.058
SpO2, %	98.00 [95.00, 100.00]	98.00 [95.00, 100.00]	98.00 [96.00, 100.00]	0.643	0.063
Hypertension, n (%)	732 (54.3)	363 (53.9)	369 (54.7)	0.785	0.018
Diabetes, n (%)	406 (30.1)	192 (28.5)	214 (31.8)	0.212	0.071
Coronary artery disease, n (%)	416 (30.9)	210 (31.2)	206 (30.6)	0.86	0.013
COPD, n (%)	170 (12.6)	89 (13.2)	81 (12.0)	0.566	0.036
Liver disease, n (%)	185 (13.7)	88 (13.1)	97 (14.4)	0.527	0.039
Atrial fibrillation (%)	234 (17.4)	119 (17.7)	115 (17.1)	0.829	0.016
Shock (%)	177 (13.1)	94 (13.9)	83 (12.3)	0.42	0.048
Sepsis (%)	935 (69.4)	473 (70.2)	462 (68.5)	0.555	0.035
Anion gap, mmol/L	15.00 [13.00, 18.00]	15.00 [13.00, 18.00]	15.00 [13.00, 18.00]	0.574	0.035
BUN, mg/dL	21.00 [14.00, 33.00]	21.00 [14.00, 34.00]	21.00 [15.00, 33.00]	0.955	0.053
Serum creatinine, mg/dL	1.00 [0.80, 1.60]	1.00 [0.80, 1.60]	1.10 [0.80, 1.58]	0.935	0.045
Serum potassium, mmol/L	4.20 [3.80, 4.70]	4.20 [3.80, 4.70]	4.20 [3.80, 4.68]	0.745	0.018
INR	1.30 [1.10, 1.50]	1.30 [1.10, 1.50]	1.30 [1.10, 1.50]	0.398	0.02
RBC, 10^{12}/L	3.65 [3.03, 4.22]	3.64 [3.04, 4.23]	3.65 [3.03, 4.21]	0.954	0.013
RDW, %	14.40 [13.40, 16.00]	14.40 [13.40, 16.10]	14.50 [13.40, 16.00]	0.553	0.005
SOFA	5.00 [3.00, 7.00]	5.00 [3.00, 8.00]	5.00 [3.00, 7.00]	0.6	0.058
GCS	14.00 [11.00, 15.00]	14.00 [11.00, 15.00]	14.00 [11.25, 15.00]	0.706	0.024
AKI KDIGO stage, n (%)				0.72	0.044
Stage 1	376 (27.9)	187 (27.7)	189 (28.0)		
Stage 2	684 (50.7)	337 (50.0)	347 (51.5)		
Stage 3	288 (21.4)	150 (22.3)	138 (20.5)		
Renal replacement therapy, n (%)	47 (3.5)	24 (3.6)	23 (3.4)	1	0.008
Vasoactive use (%)	501 (37.2)	256 (38.0)	245 (36.4)	0.573	0.034
Ventilator use (%)	624 (46.3)	317 (47.0)	307 (45.5)	0.623	0.03

Abbreviations: SBP Systolic blood pressure, DBP Diastolic blood pressure, SpO2 percutaneous oxygen saturation, COPD chronic obstructive pulmonary disease, BUN Blood urea nitrogen, INR international normalized ratio, RBC red blood count, RDW red cell distribution width, SOFA Sequential Organ Failure Assessment, GCS Glasgow Coma Scale, KDIGO Kidney Disease: Improving Global

Outcomes. Normally distributed data are presented as the mean (SD) (analysis of variance); non-normally distributed data are presented as median (IQR) (nonparametric Wilcoxon test); and categorical variables are presented as n (%) (chi-square test).

Supplemental Table 2. Original data of the patients included in the study

subject_id	hadm_id	stay_id	drug	starttime	stoptime
10001884	26184834	37510196	Amoxicillin-	2131/1/9 11:00	2131/1/11 05:00
10001884	26184834	37510196	Amoxicillin-	2131/1/11 10:00	2131/1/11 09:00
10001884	26184834	37510196	Amoxicillin-	2131/1/12 11:00	2131/1/18 11:00
10002495	24982426	36753294	Amoxicillin	2141/5/22 23:00	2141/5/23 08:00
10007677	27180483	34992648	Amoxicillin	2120/11/23 18:00	2120/11/25 12:00
10007677	27180483	34992648	Amoxicillin	2120/11/25 18:00	2120/11/25 15:00
10007677	27180483	34992648	Amoxicillin	2120/11/25 20:00	2120/11/26 19:00
10019957	28761725	37708849	Amoxicillin-	2135/11/14 19:00	2135/11/15 22:00
10029057	28919637	36004746	Amoxicillin	2187/8/2 14:00	2187/8/2 13:00
10029057	28919637	36004746	Amoxicillin	2187/8/11 10:00	2187/8/11 19:00
10029057	28919637	36004746	Amoxicillin-	2187/8/6 16:00	2187/8/6 13:00
10029057	28919637	36004746	Amoxicillin-	2187/8/6 16:00	2187/8/8 11:00
10048723	25762910	36542901	Amoxicillin-	2185/6/15 12:00	2185/6/15 19:00
10054716	25339060	33668354	Amoxicillin-	2136/6/2 09:00	2136/6/3 18:00
10057731	29234056	30879445	Amoxicillin-	2156/1/1 15:00	2156/1/2 23:00
10060531	27040798	36935478	Amoxicillin-	2153/5/28 20:00	2153/5/28 22:00
10080961	24032231	39385708	Amoxicillin-	2140/2/29 17:00	2140/3/1 21:00
10081525	28566281	33832369	Amoxicillin-	2148/2/1 17:00	2148/2/4 19:00
10086022	24567350	34048359	Amoxicillin-	2159/11/18 17:00	2159/11/20 20:00
10104549	25502861	32140809	Amoxicillin	2202/3/2 12:00	2202/3/4 20:00
10109150	24071719	36142655	Amoxicillin	2148/6/7 19:00	2148/6/8 12:00
10114694	22418467	35032951	Amoxicillin-	2163/4/2 20:00	2163/4/2 21:00
10127469	23079904	38026241	Amoxicillin-	2162/9/11 10:00	2162/9/12 18:00
10128085	27136294	39990093	Amoxicillin-	2145/8/29 15:00	2145/9/1 22:00
10145417	27257164	34490961	Amoxicillin-	2129/11/4 17:00	2129/11/6 19:00
10148145	28234938	39196630	Amoxicillin-	2162/10/1 19:00	2162/10/2 18:00
10157454	27280709	38216308	Amoxicillin-	2182/5/8 14:00	2182/5/15 13:00
10157454	27280709	38216308	Amoxicillin-	2182/5/15 14:00	2182/5/16 10:00
10180134	27497725	33413600	Amoxicillin	2137/5/4 17:00	2137/5/5 13:00
10184327	28576249	35263906	Amoxicillin	2132/1/7 11:00	2132/1/7 21:00
10198809	26596379	36751608	Amoxicillin-	2142/5/25 09:00	2142/5/26 23:00
10199297	20665998	30860531	AMOXicillin	2172/10/5 20:00	2172/10/7 21:00
10242601	26646532	33609202	Amoxicillin-	2153/1/19 17:00	2153/1/20 19:00
10251262	26787243	32990090	Amoxicillin	2185/2/28 08:00	2185/2/28 21:00
10254956	21021888	34532082	Amoxicillin-	2178/2/9 11:00	2178/2/10 20:00
10263098	29438982	32118761	Amoxicillin-	2154/9/20 20:00	2154/9/22 20:00
10274857	28804667	31587707	Amoxicillin-	2157/3/27 09:00	2157/3/27 22:00
10277119	21412538	31235375	Amoxicillin-	2182/10/19 20:00	2182/10/22 02:00
10283863	27425584	34162009	Amoxicillin-	2161/5/1 11:00	2161/5/4 19:00
10286972	21106212	35884839	Amoxicillin-	2163/4/17 15:00	2163/4/17 19:00
10292598	27780489	33692761	Amoxicillin-	2163/5/26 11:00	2163/6/1 17:00
10293741	27441207	33024805	Amoxicillin-	2176/7/12 20:00	2176/7/15 23:00
10298894	28979197	34583194	Amoxicillin-	2129/5/20 13:00	2129/5/22 19:00
10301071	25678968	34850631	Amoxicillin	2183/12/31 15:00	2183/12/31 19:00
10314252	25055534	32831765	Amoxicillin-	2165/10/8 13:00	2165/10/10 19:00

10368327	29867223	30946246	Amoxicillin-	2151/2/18 21:00	2151/2/18 20:00
10368327	29867223	30946246	Amoxicillin-	2151/2/18 21:00	2151/2/24 23:00
10368757	29854960	31301515	Amoxicillin-	2114/2/18 19:00	2114/2/19 23:00
10379240	29438229	37094692	Amoxicillin-	2137/5/13 10:00	2137/5/13 20:00
10384160	27721508	32591955	Amoxicillin-	2178/2/3 15:00	2178/2/7 02:00
10411588	23766173	34773422	Amoxicillin-	2126/4/26 18:00	2126/4/28 21:00
10413295	22438881	37973258	Amoxicillin	2170/12/29 21:00	2171/1/7 19:00
10413379	29187152	39857255	Amoxicillin-	2130/8/5 20:00	2130/8/6 21:00
10429729	26929922	36717130	Amoxicillin-	2123/1/13 19:00	2123/1/16 22:00
10444484	27161321	31924428	Amoxicillin-	2180/5/10 08:00	2180/5/10 20:00
10449408	22698294	36257805	Amoxicillin	2141/12/20 14:00	2141/12/21 11:00
10452075	28206315	38011424	Amoxicillin	2111/4/9 10:00	2111/4/13 22:00
10453488	28720667	31161420	Amoxicillin-	2150/8/25 12:00	2150/8/26 20:00
10471469	24537345	31395309	Amoxicillin	2161/5/11 08:00	2161/5/10 09:00
10474329	25966222	31771936	Amoxicillin	2139/6/24 19:00	2139/6/28 18:00
10481270	20529890	31304792	Amoxicillin-	2155/8/10 08:00	2155/8/12 19:00
10496352	20886029	32891161	Amoxicillin-	2117/1/23 09:00	2117/1/23 18:00
10511536	28205412	31934149	Amoxicillin-	2169/5/4 09:00	2169/5/4 18:00
10527186	25241870	36965690	Amoxicillin-	2191/11/30 11:00	2191/11/30 12:00
10527186	25241870	36965690	Amoxicillin-	2191/12/8 18:00	2191/12/9 22:00
10553843	20730479	38712799	Amoxicillin-	2115/4/12 10:00	2115/4/12 21:00
10556002	29077178	36148150	Amoxicillin	2160/9/23 12:00	2160/9/24 12:00
10583751	25396614	39774274	Amoxicillin	2127/6/11 17:00	2127/6/12 21:00
10598185	29740308	37935156	Amoxicillin-	2190/4/4 20:00	2190/4/4 21:00
10598185	29740308	37935156	Amoxicillin-	2190/4/7 20:00	2190/4/8 23:00
10671800	29192269	30656080	Amoxicillin-	2148/7/21 22:00	2148/7/22 20:00
10671800	29192269	30656080	Amoxicillin-	2148/7/22 22:00	2148/7/27 23:00
10689166	28768474	34539026	Amoxicillin-	2120/2/15 12:00	2120/2/16 20:00
10701573	24245464	36827393	Amoxicillin	2180/8/26 18:00	2180/8/29 10:00
10701573	24245464	36827393	Amoxicillin	2180/8/29 18:00	2180/8/31 19:00
10719051	24805218	32639802	Amoxicillin-	2197/9/24 12:00	2197/9/25 23:00
10723529	29196823	32013282	Amoxicillin-	2122/1/2 13:00	2122/1/2 18:00
10739820	23044966	37175483	Amoxicillin	2182/11/6 08:00	2182/11/6 07:00
10739820	23044966	37175483	Amoxicillin	2182/11/7 08:00	2182/11/6 10:00
10739820	23044966	37175483	Amoxicillin	2182/11/7 08:00	2182/11/9 09:00
10754405	20972164	38037508	Amoxicillin	2165/9/12 14:00	2165/9/12 22:00
10758777	23334577	32284012	Amoxicillin	2185/2/9 16:00	2185/2/11 18:00
10763729	29936065	36052907	Amoxicillin-	2181/3/21 12:00	2181/3/29 08:00
10763729	29936065	36052907	Amoxicillin-	2181/3/29 12:00	2181/3/30 10:00
10792544	21078377	30541650	Amoxicillin-	2113/10/26 09:00	2113/10/27 19:00
10813295	22053636	32180163	Amoxicillin-	2124/11/21 18:00	2124/11/22 21:00
10815669	29343447	35145757	Amoxicillin-	2177/7/6 10:00	2177/7/7 23:00
10819468	25785127	30579374	Amoxicillin	2166/10/2 14:00	2166/10/9 13:00
10832658	20617215	38291404	Amoxicillin-	2188/10/20 00:00	2188/10/22 21:00
10857996	21701924	34678772	AMOXicillin	2136/1/22 14:00	2136/1/27 20:00
10864544	20673052	33871289	Amoxicillin-	2114/7/2 08:00	2114/7/1 23:00

10866370	21405297	38853401	Amoxicillin	2114/4/9 14:00	2114/4/12 12:00
10875292	25385589	30325487	Amoxicillin-	2126/10/2 12:00	2126/10/3 16:00
10875292	25385589	30325487	Amoxicillin-	2126/10/4 00:00	2126/10/6 15:00
10881070	25103836	36370324	Amoxicillin-	2153/11/28 11:00	2153/12/3 19:00
10890618	27058658	34758144	Amoxicillin-	2133/7/9 14:00	2133/7/10 19:00
10913302	22678858	32328501	Amoxicillin	2193/8/2 13:00	2193/8/2 13:00
10913302	22678858	32328501	Amoxicillin	2193/8/3 01:00	2193/8/6 22:00
10923152	26619736	30382119	Amoxicillin-	2116/1/1 12:00	2116/1/2 17:00
10939051	21436622	37930768	Amoxicillin-	2136/11/17 08:00	2136/11/18 17:00
10993119	29991038	34667520	Amoxicillin-	2136/3/28 17:00	2136/3/29 09:00
10993119	29991038	34667520	Amoxicillin-	2136/3/30 14:00	2136/4/2 21:00
10993119	29991038	34667520	Amoxicillin-	2136/4/2 22:00	2136/4/5 21:00
11017350	24993015	31984809	Amoxicillin-	2175/5/24 11:00	2175/5/25 18:00
11023526	24714149	30328075	Amoxicillin	2178/6/30 09:00	2178/6/30 15:00
11024917	25998301	35606458	Amoxicillin-	2136/11/6 09:00	2136/11/13 22:00
11033150	27209360	33739064	Amoxicillin-	2133/10/19 20:00	2133/10/22 14:00
11033150	27209360	33739064	Amoxicillin-	2133/10/22 10:00	2133/10/25 13:00
11050359	29779708	33640350	Amoxicillin-	2130/2/22 19:00	2130/2/27 04:00
11055094	23118700	39770803	Amoxicillin	2153/4/16 11:00	2153/4/18 21:00
11057464	23467550	33868624	AMOXicillin	2165/3/26 08:00	2165/4/5 21:00
11057464	23467550	33868624	AMOXicillin	2165/3/26 20:00	2165/3/28 21:00
11065164	29715671	30379732	Amoxicillin	2167/2/18 17:00	2167/2/18 17:00
11065164	29715671	30379732	Amoxicillin	2167/2/18 17:00	2167/2/19 16:00
11065164	29715671	30379732	Amoxicillin	2167/2/24 03:00	2167/2/25 10:00
11065164	29715671	30379732	Amoxicillin	2167/2/24 15:00	2167/2/24 21:00
11065164	29715671	30379732	Amoxicillin	2167/2/25 15:00	2167/2/26 11:00
11065164	29715671	30379732	AMOXicillin	2167/2/26 20:00	2167/3/1 23:00
11087224	29975017	34836186	Amoxicillin-	2128/1/6 12:00	2128/1/8 19:00
11087410	25148713	31593726	Amoxicillin-	2148/1/11 13:00	2148/1/11 19:00
11091187	26857576	32318405	Amoxicillin	2168/11/30 20:00	2168/12/3 23:00
11096768	27422643	30520948	Amoxicillin-	2149/1/7 08:00	2149/1/10 15:00
11121168	26907315	33950371	Amoxicillin-	2198/5/23 08:00	2198/5/23 17:00
11171757	25970749	31158591	Amoxicillin-	2150/11/30 08:00	2150/11/30 09:00
11171757	25970749	31158591	Amoxicillin-	2150/11/30 20:00	2150/11/30 20:00
11189964	26650355	36861501	Amoxicillin	2134/12/24 17:00	2134/12/25 10:00
11190737	21310093	37967605	Amoxicillin	2189/4/1 13:00	2189/4/4 19:00
11200955	23271346	35372416	Amoxicillin	2194/9/23 18:00	2194/9/24 10:00
11200955	23271346	35372416	Amoxicillin	2194/9/24 11:00	2194/9/27 10:00
11239107	20342738	36124853	Amoxicillin-	2112/11/30 20:00	2112/12/5 17:00
11249665	25042938	33382673	Amoxicillin	2132/5/9 14:00	2132/5/10 15:00
11249665	25042938	33382673	Amoxicillin	2132/5/12 10:00	2132/5/18 13:00
11249860	29859525	36913502	AMOXicillin	2176/6/12 20:00	2176/6/15 09:00
11260884	21316796	32514082	Amoxicillin-	2200/1/27 20:00	2200/1/29 22:00
11283455	23242179	35933450	Amoxicillin-	2124/6/1 13:00	2124/6/3 19:00
11352800	28344050	38301645	Amoxicillin	2133/11/30 15:00	2133/11/30 22:00
11352800	28344050	38301645	Amoxicillin	2133/11/30 23:00	2133/12/4 22:00

11367185	21999645	31586765	Amoxicillin-	2138/6/24 12:00	2138/6/25 20:00
11399516	24843518	39422318	Amoxicillin-	2148/8/25 10:00	2148/8/25 17:00
11408401	20915702	33753561	Amoxicillin-	2182/6/13 12:00	2182/6/13 20:00
11430311	25074223	30184955	Amoxicillin-	2126/11/5 04:00	2126/11/8 13:00
11437855	25122978	38711627	Amoxicillin-	2115/10/18 22:00	2115/10/19 08:00
11449299	24466693	38066884	Amoxicillin-	2136/1/8 08:00	2136/1/8 20:00
11459626	23315647	30306711	Amoxicillin-	2160/11/25 11:00	2160/12/2 12:00
11468840	27111775	30274311	Amoxicillin-	2184/4/12 11:00	2184/4/13 22:00
11510549	21791514	30187936	Amoxicillin	2172/3/5 22:00	2172/3/7 19:00
11510549	21791514	30187936	Amoxicillin	2172/3/7 20:00	2172/3/8 19:00
11512695	26518072	39063436	Amoxicillin-	2175/1/3 09:00	2175/1/3 18:00
11527001	24988229	39024324	Amoxicillin	2158/7/19 14:00	2158/7/20 01:00
11546715	28446695	35619929	Amoxicillin-	2147/9/5 11:00	2147/9/5 18:00
11548284	24670416	30117693	Amoxicillin-	2176/7/6 14:00	2176/7/9 21:00
11557186	22752628	35469955	Amoxicillin-	2167/12/10 08:00	2167/12/9 10:00
11564481	29255648	33651934	Amoxicillin-	2158/2/17 14:00	2158/2/19 15:00
11564481	29255648	33651934	Amoxicillin-	2158/2/21 08:00	2158/2/22 22:00
11564836	23167844	39138675	Amoxicillin-	2189/9/24 13:00	2189/9/24 20:00
11573679	20863501	30525898	Amoxicillin-	2183/4/11 14:00	2183/4/14 12:00
11573679	20863501	30525898	Amoxicillin-	2183/4/14 14:00	2183/4/15 08:00
11577761	26540364	36043309	Amoxicillin-	2130/5/25 10:00	2130/5/25 17:00
11579903	25509560	36450265	Amoxicillin-	2163/7/13 09:00	2163/7/14 09:00
11589067	23073228	36329345	Amoxicillin-	2136/6/29 16:00	2136/7/1 17:00
11593664	24141348	30954111	Amoxicillin	2144/5/13 13:00	2144/5/15 21:00
11595727	21528129	31876858	Amoxicillin-	2139/11/1 09:00	2139/11/2 17:00
11595745	26877531	38728778	Amoxicillin-	2196/11/22 14:00	2196/11/23 20:00
11600106	24964764	35918303	Amoxicillin-	2199/8/14 13:00	2199/8/18 19:00
11601773	20860481	31594387	Amoxicillin-	2168/2/15 11:00	2168/2/16 12:00
11601773	20860481	31594387	Amoxicillin-	2168/2/16 13:00	2168/2/16 21:00
11663663	29081759	37010167	Amoxicillin-	2152/8/24 13:00	2152/8/25 23:00
11667451	21081277	33975326	Amoxicillin-	2123/8/13 12:00	2123/8/13 20:00
11675574	29793379	35295874	Amoxicillin-	2163/1/12 10:00	2163/1/13 22:00
11675574	29793379	35295874	Amoxicillin-	2163/1/13 20:00	2163/1/15 08:00
11675574	29793379	35295874	Amoxicillin-	2163/1/15 20:00	2163/1/16 00:00
11686464	23428361	38331996	Amoxicillin-	2124/4/4 10:00	2124/4/4 19:00
11691384	20500465	35264055	Amoxicillin-	2166/9/24 12:00	2166/9/25 16:00
11728785	25801255	34980648	Amoxicillin-	2138/5/5 09:00	2138/5/5 00:00
11747667	24337698	38064970	Amoxicillin-	2115/1/12 17:00	2115/1/16 11:00
11747893	20563272	32344065	Amoxicillin-	2119/10/26 12:00	2119/11/2 08:00
11747893	20563272	32344065	Amoxicillin-	2119/11/2 09:00	2119/11/3 10:00
11747893	20563272	32344065	Amoxicillin-	2119/11/5 12:00	2119/11/23 18:00
11747893	20563272	32344065	Amoxicillin-	2119/11/26 13:00	2119/11/26 16:00
11832757	20204340	36690574	Amoxicillin-	2153/3/27 17:00	2153/3/28 23:00
11836722	22659832	32542144	Amoxicillin-	2160/8/16 20:00	2160/8/19 21:00
11842194	29659910	30580160	Amoxicillin-	2171/11/15 16:00	2171/11/17 15:00
11855159	27842026	36798409	Amoxicillin-	2166/2/26 08:00	2166/3/1 21:00

11858684	24200796	38945023	Amoxicillin-	2122/10/20 08:00	2122/10/22 21:00
11895151	28452803	36147922	Amoxicillin	2181/2/24 12:00	2181/2/27 18:00
11919347	28606369	39156401	Amoxicillin-	2196/8/27 19:00	2196/8/29 19:00
11919347	28606369	39156401	Amoxicillin-	2196/8/29 07:00	2196/9/1 09:00
11919347	28606369	39156401	Amoxicillin-	2196/9/1 19:00	2196/9/1 09:00
11919347	28606369	39156401	Amoxicillin-	2196/9/1 19:00	2196/9/3 14:00
11919347	28606369	39156401	Amoxicillin-	2196/9/3 15:00	2196/9/7 10:00
11921391	21048337	34511936	Amoxicillin	2122/2/21 19:00	2122/2/23 10:00
11921391	21048337	34511936	Amoxicillin-	2122/2/23 11:00	2122/2/24 18:00
11921391	21048337	34511936	Amoxicillin-	2122/2/24 19:00	2122/2/26 13:00
11921391	21048337	34511936	Amoxicillin-	2122/2/26 19:00	2122/2/27 06:00
11924230	25215676	38110754	Amoxicillin	2151/7/31 11:00	2151/7/31 10:00
11933892	24379230	35913304	Amoxicillin	2144/2/13 11:00	2144/2/14 12:00
11933892	24379230	35913304	Amoxicillin	2144/2/14 23:00	2144/2/24 20:00
11947833	24818125	33740171	Amoxicillin-	2120/4/21 18:00	2120/4/22 16:00
11947833	24818125	33740171	Amoxicillin-	2120/4/23 06:00	2120/4/25 05:00
11962176	22847498	34999626	Amoxicillin-	2204/4/6 21:00	2204/4/10 21:00
11971799	23999593	32478563	Amoxicillin-	2162/10/18 12:00	2162/10/18 23:00
11980812	20346878	34036698	Amoxicillin	2155/12/13 10:00	2155/12/16 15:00
11980812	20346878	34036698	Amoxicillin	2155/12/16 16:00	2155/12/17 12:00
11994020	23379501	35029438	Amoxicillin-	2131/11/13 08:00	2131/11/13 14:00
11994020	23379501	35029438	Amoxicillin-	2131/11/14 20:00	2131/11/16 19:00
12006207	25368862	30275454	Amoxicillin-	2124/3/13 18:00	2124/3/15 20:00
12051541	29560990	35741536	Amoxicillin-	2148/9/19 11:00	2148/9/19 15:00
12060567	29077318	39021590	Amoxicillin-	2124/10/13 14:00	2124/10/14 22:00
12078802	23360195	34244028	Amoxicillin-	2186/3/16 12:00	2186/3/18 09:00
12080183	29569040	30663943	Amoxicillin-	2162/1/1 20:00	2162/1/3 21:00
12135369	24204652	38242652	Amoxicillin-	2174/1/31 14:00	2174/1/31 20:00
12138637	23008526	31434650	Amoxicillin-	2162/9/30 20:00	2162/10/1 13:00
12138637	23008526	31434650	Amoxicillin-	2162/10/3 10:00	2162/10/3 10:00
12140206	26782912	30281960	Amoxicillin-	2143/5/17 08:00	2143/5/16 19:00
12153100	20372597	34358848	Amoxicillin-	2165/1/25 12:00	2165/1/28 11:00
12153100	20372597	34358848	Amoxicillin-	2165/1/29 08:00	2165/1/29 14:00
12153312	26352325	35483053	Amoxicillin-	2141/10/2 21:00	2141/10/3 12:00
12153312	26352325	35483053	Amoxicillin-	2141/10/3 13:00	2141/10/6 20:00
12200381	27944373	33069949	Amoxicillin	2146/7/4 16:00	2146/7/8 11:00
12213713	26670521	36696382	Amoxicillin-	2130/9/9 08:00	2130/9/11 21:00
12219559	28621241	34375944	Amoxicillin	2147/10/25 11:00	2147/10/25 11:00
12232906	22837428	33817997	Amoxicillin	2175/6/30 06:00	2175/7/1 23:00
12244299	28938467	39919566	Amoxicillin-	2110/11/19 16:00	2110/11/20 09:00
12255996	20288522	36718729	Amoxicillin-	2172/11/12 11:00	2172/11/13 17:00
12263437	28665712	38502100	Amoxicillin-	2148/10/20 20:00	2148/10/26 01:00
12273109	22625853	34916614	Amoxicillin-	2136/12/17 20:00	2136/12/21 00:00
12277308	24450048	30741101	Amoxicillin-	2172/11/20 14:00	2172/11/23 14:00
12277308	24450048	30741101	Amoxicillin-	2172/11/24 02:00	2172/11/24 17:00
12277308	24450048	30741101	Amoxicillin-	2172/11/25 20:00	2172/12/2 16:00

12279729	22579957	39146539	Amoxicillin	2157/7/14 11:00	2157/7/14 12:00
12279729	22579957	39146539	Amoxicillin	2157/7/14 23:00	2157/7/21 23:00
12296616	29042115	39348430	Amoxicillin	2167/10/21 13:00	2167/10/25 19:00
12323655	22488105	31193470	Amoxicillin-	2146/6/22 10:00	2146/6/22 19:00
12328902	29372998	31597943	Amoxicillin	2177/1/31 16:00	2177/2/1 12:00
12337452	27006668	30346033	Amoxicillin-	2154/7/15 11:00	2154/7/16 23:00
12351810	28611290	36482405	Amoxicillin-	2157/12/14 11:00	2157/12/14 19:00
12355662	28885970	38077771	Amoxicillin	2114/4/2 12:00	2114/4/2 15:00
12355662	28885970	38077771	Amoxicillin	2114/4/3 08:00	2114/4/7 13:00
12355662	28885970	38077771	Amoxicillin	2114/4/7 16:00	2114/4/10 18:00
12389435	20586024	39961471	Amoxicillin-	2149/10/20 09:00	2149/10/21 16:00
12402843	26830831	34375647	Amoxicillin-	2189/8/3 09:00	2189/8/10 15:00
12411239	23028159	37646717	Amoxicillin-	2163/3/26 19:00	2163/3/29 17:00
12416498	20695760	33091044	Amoxicillin	2153/7/8 02:00	2153/7/7 20:00
12437680	28617744	31262924	Amoxicillin-	2183/5/21 23:00	2183/5/22 17:00
12443606	21198311	36306727	Amoxicillin	2146/1/1 11:00	2146/1/1 20:00
12444275	20076874	31896252	Amoxicillin-	2176/3/9 13:00	2176/3/10 09:00
12444275	20076874	31896252	Amoxicillin-	2176/3/10 10:00	2176/3/11 12:00
12455298	27900642	30361048	Amoxicillin-	2189/11/2 02:00	2189/11/2 19:00
12458978	25268144	31781982	Amoxicillin-	2172/10/5 15:00	2172/10/8 00:00
12464172	20245963	30496982	Amoxicillin	2158/10/25 19:00	2158/10/28 09:00
12464172	20245963	30496982	AMOXicillin	2158/10/28 16:00	2158/10/28 21:00
12468016	21026864	33038289	Amoxicillin-	2130/6/26 09:00	2130/6/28 17:00
12500183	20119491	39634568	Amoxicillin-	2119/6/19 14:00	2119/6/20 16:00
12517153	20921668	36838634	Amoxicillin-	2186/6/17 12:00	2186/6/19 18:00
12532638	28562533	34410542	Amoxicillin	2184/1/19 13:00	2184/1/23 14:00
12532638	28562533	34410542	Amoxicillin	2184/1/28 08:00	2184/1/28 17:00
12542123	22606140	33730740	Amoxicillin	2157/1/30 11:00	2157/1/31 02:00
12542123	22606140	33730740	Amoxicillin	2157/1/31 10:00	2157/1/31 10:00
12544783	28690566	36727026	Amoxicillin-	2180/5/6 16:00	2180/5/13 16:00
12579712	25200745	36444505	Amoxicillin-	2185/1/19 10:00	2185/1/21 18:00
12620822	23412935	34292607	Amoxicillin	2186/9/27 00:00	2186/10/7 16:00
12620822	23412935	34292607	Amoxicillin	2186/10/7 12:00	2186/10/8 02:00
12641622	25607164	39919258	Amoxicillin-	2184/6/17 13:00	2184/6/19 22:00
12653468	21250157	32216357	Amoxicillin	2193/2/11 14:00	2193/2/11 22:00
12655454	29663271	35565234	Amoxicillin-	2177/11/21 14:00	2177/11/23 09:00
12661994	27356989	38091853	Amoxicillin-	2176/3/16 08:00	2176/3/18 12:00
12691447	25925370	33713518	Amoxicillin	2117/8/17 19:00	2117/8/21 18:00
12694726	21025963	34468417	Amoxicillin	2161/6/16 09:00	2161/6/16 11:00
12697173	21607700	31370142	Amoxicillin	2136/5/8 17:00	2136/5/8 21:00
12729806	20571804	32497054	Amoxicillin-	2133/7/31 16:00	2133/8/7 15:00
12730126	25754400	36204955	Amoxicillin-	2181/3/5 10:00	2181/3/5 18:00
12730126	25754400	36204955	Amoxicillin-	2181/3/5 20:00	2181/3/8 18:00
12751607	27373350	37987126	Amoxicillin-	2188/7/18 16:00	2188/7/23 03:00
12770182	20446666	34901199	Amoxicillin-	2110/1/19 00:00	2110/1/19 06:00
12793412	24077990	32651382	Amoxicillin-	2147/12/16 15:00	2147/12/19 17:00

12805811	28354605	31976217	Amoxicillin-	2170/1/4 15:00	2170/1/5 17:00
12816177	27139242	30044542	Amoxicillin	2133/4/30 12:00	2133/5/13 12:00
12837948	20012163	34435255	Amoxicillin	2147/1/10 14:00	2147/1/12 23:00
12855476	24121938	32633893	Amoxicillin-	2157/11/28 09:00	2157/11/30 14:00
12857645	25368085	39771268	Amoxicillin-	2145/9/16 20:00	2145/9/17 21:00
12883163	22554993	35972126	Amoxicillin-	2136/5/26 12:00	2136/5/27 08:00
12899504	22935898	33296284	Amoxicillin	2137/11/21 11:00	2137/11/30 21:00
12942397	25572412	31959330	Amoxicillin-	2151/3/27 14:00	2151/3/28 08:00
12943458	29587220	33884935	Amoxicillin-	2165/1/9 11:00	2165/1/9 21:00
12945639	28006697	38087113	Amoxicillin-	2116/4/27 11:00	2116/4/30 16:00
12953330	23826002	33934932	Amoxicillin	2157/4/5 08:00	2157/4/4 15:00
12953330	23826002	33934932	Amoxicillin	2157/4/5 08:00	2157/4/8 02:00
12972442	21188097	31715771	Amoxicillin-	2189/8/5 13:00	2189/8/6 15:00
12974332	25845436	30720284	Amoxicillin-	2131/1/10 15:00	2131/1/14 18:00
12981244	24929566	37327428	Amoxicillin-	2152/7/29 08:00	2152/7/29 09:00
12981244	24929566	37327428	Amoxicillin-	2152/7/30 08:00	2152/7/30 23:00
12988934	22410634	31935640	Amoxicillin	2119/2/19 14:00	2119/2/20 13:00
12991778	27345788	31289002	Amoxicillin-	2183/4/8 18:00	2183/4/12 15:00
13030403	20259761	33838516	Amoxicillin-	2187/7/1 08:00	2187/7/1 11:00
13030403	20259761	33838516	Amoxicillin-	2187/7/1 23:00	2187/7/2 09:00
13060492	26905306	32675533	Amoxicillin-	2161/9/18 09:00	2161/9/20 11:00
13069434	26792980	31611273	Amoxicillin	2171/9/27 08:00	2171/9/28 15:00
13069434	26792980	31611273	Amoxicillin	2171/9/28 08:00	2171/9/30 19:00
13074187	29377961	36569622	AMOXicillin	2193/4/25 05:00	2193/4/27 21:00
13098061	25183929	34077257	Amoxicillin-	2119/9/7 20:00	2119/9/7 21:00
13102222	22865835	34094273	Amoxicillin-	2128/7/30 07:00	2128/7/30 12:00
13102222	22865835	34094273	Amoxicillin-	2128/8/7 12:00	2128/8/7 19:00
13109130	20087737	30056626	Amoxicillin-	2168/9/15 09:00	2168/9/15 19:00
13109130	20087737	30056626	Amoxicillin-	2168/9/17 15:00	2168/9/18 19:00
13110443	25130175	39714859	Amoxicillin-	2191/11/16 14:00	2191/11/17 11:00
13112514	29438586	35660191	Amoxicillin-	2111/8/17 17:00	2111/8/18 21:00
13112514	29438586	35660191	Amoxicillin-	2111/8/17 20:00	2111/8/17 16:00
13122945	22242120	30145592	Amoxicillin-	2138/2/24 20:00	2138/2/25 08:00
13131863	20059368	34125384	Amoxicillin-	2206/8/28 13:00	2206/8/29 19:00
13132402	23239423	31485989	Amoxicillin	2112/9/17 13:00	2112/9/19 17:00
13142963	29235745	34194516	Amoxicillin-	2156/3/20 16:00	2156/3/20 22:00
13144697	28257074	37714884	Amoxicillin-	2134/2/8 13:00	2134/2/10 18:00
13155939	25316345	39233714	Amoxicillin	2189/5/25 18:00	2189/5/28 18:00
13159019	24067663	30802766	Amoxicillin-	2139/11/14 20:00	2139/11/14 20:00
13160119	21234259	34029552	Amoxicillin-	2137/7/26 22:00	2137/7/27 09:00
13169821	24434590	33715280	Amoxicillin-	2176/5/2 13:00	2176/5/4 23:00
13180512	24527016	38246117	Amoxicillin-	2151/4/29 20:00	2151/5/4 07:00
13180512	24527016	38246117	Amoxicillin-	2151/5/4 08:00	2151/5/5 07:00
13187609	22681948	37707849	Amoxicillin-	2119/2/21 20:00	2119/2/25 19:00
13196638	25702185	37519903	Amoxicillin-	2132/7/29 10:00	2132/7/31 22:00
13209752	21771189	36623230	Amoxicillin-	2165/12/10 09:00	2165/12/11 07:00

13224505	25504165	33046699	Amoxicillin-	2175/2/20 13:00	2175/2/20 19:00
13224505	25504165	33046699	Amoxicillin-	2175/2/20 20:00	2175/2/21 20:00
13225587	24705847	39691126	Amoxicillin-	2125/10/4 13:00	2125/10/6 21:00
13228563	25432751	38497191	Amoxicillin-	2133/7/9 15:00	2133/7/12 08:00
13228563	25432751	38497191	Amoxicillin-	2133/7/12 15:00	2133/7/13 20:00
13228563	25432751	38497191	Amoxicillin-	2133/7/12 20:00	2133/7/13 20:00
13245622	20487825	34716178	Amoxicillin	2158/10/10 00:00	2158/10/10 12:00
13245622	20487825	34716178	Amoxicillin	2158/10/10 16:00	2158/10/12 18:00
13280145	25038913	30352811	Amoxicillin-	2165/6/18 20:00	2165/6/18 13:00
13280145	25038913	30352811	Amoxicillin-	2165/6/18 20:00	2165/6/19 20:00
13280145	25038913	30352811	Amoxicillin-	2165/6/19 20:00	2165/6/21 19:00
13292682	29699752	39841435	Amoxicillin-	2154/2/4 08:00	2154/2/3 23:00
13292682	29699752	39841435	Amoxicillin-	2154/2/4 08:00	2154/2/8 18:00
13299672	22499620	34916121	Amoxicillin-	2120/7/28 13:00	2120/7/29 14:00
13313907	28672917	37092980	Amoxicillin-	2170/8/11 10:00	2170/8/12 18:00
13350724	26551913	33991123	Amoxicillin-	2135/12/12 07:00	2135/12/13 10:00
13351112	28345415	31840044	Amoxicillin-	2159/9/1 20:00	2159/9/4 11:00
13352295	23350373	34368960	Amoxicillin-	2136/6/6 13:00	2136/6/6 18:00
13364281	27693656	38887955	Amoxicillin-	2170/2/9 10:00	2170/2/15 21:00
13405183	27932719	32894104	Amoxicillin-	2165/7/22 11:00	2165/7/24 10:00
13411396	22726951	39683516	Amoxicillin-	2163/4/22 15:00	2163/4/22 21:00
13411396	22726951	39683516	Amoxicillin-	2163/4/23 17:00	2163/4/25 07:00
13411396	22726951	39683516	Amoxicillin-	2163/4/25 17:00	2163/4/27 16:00
13413962	23659372	35624878	Amoxicillin-	2146/7/19 20:00	2146/7/20 23:00
13422454	28945493	35067960	Amoxicillin-	2139/5/31 08:00	2139/5/31 20:00
13422599	27939657	30312349	Amoxicillin-	2182/11/27 09:00	2182/11/30 16:00
13425635	23951171	31936912	Amoxicillin-	2146/3/7 12:00	2146/3/9 19:00
13434974	24647970	37463866	Amoxicillin	2151/11/21 08:00	2151/11/23 19:00
13442558	22665778	30311657	Amoxicillin	2114/6/23 15:00	2114/6/23 16:00
13442558	22665778	30311657	Amoxicillin-	2114/6/24 10:00	2114/6/28 09:00
13469476	20113297	37054283	Amoxicillin-	2112/2/2 15:00	2112/2/3 22:00
13476942	25516569	34876524	Amoxicillin-	2186/11/12 01:00	2186/11/14 00:00
13477106	28187845	33948500	Amoxicillin-	2177/10/27 12:00	2177/10/30 06:00
13505628	26682645	31983448	Amoxicillin	2138/8/31 18:00	2138/9/5 17:00
13511021	22431215	35569430	Amoxicillin-	2158/10/13 09:00	2158/10/16 19:00
13514465	28109699	36277397	Amoxicillin-	2120/11/6 09:00	2120/11/7 21:00
13515075	26441595	30281139	Amoxicillin-	2112/12/20 20:00	2112/12/21 19:00
13552577	26690457	34314883	Amoxicillin-	2134/5/21 20:00	2134/5/22 14:00
13552577	26690457	34314883	Amoxicillin-	2134/5/22 20:00	2134/5/23 09:00
13565628	21266167	32239611	Amoxicillin-	2176/7/2 11:00	2176/7/7 17:00
13586936	22114434	37047554	Amoxicillin-	2128/6/25 12:00	2128/6/25 18:00
13590974	29465095	34634701	Amoxicillin-	2171/11/28 16:00	2171/12/2 03:00
13618741	24403199	30400058	Amoxicillin	2119/3/6 20:00	2119/3/14 00:00
13641406	26082694	32921448	Amoxicillin	2135/9/8 19:00	2135/9/11 18:00
13647229	27726944	38144823	Amoxicillin-	2113/5/10 23:00	2113/5/11 09:00
13671107	22907089	31185256	Amoxicillin-	2142/7/15 20:00	2142/7/16 19:00

13671107	22907089	31185256	Amoxicillin-	2142/7/16 20:00	2142/7/20 19:00
13684752	27196810	36436673	Amoxicillin	2129/4/23 17:00	2129/4/28 20:00
13694166	22659941	37107409	Amoxicillin-	2155/6/22 15:00	2155/6/22 22:00
13699064	23168384	34268304	Amoxicillin-	2113/2/23 12:00	2113/2/26 21:00
13719117	25891727	39374154	Amoxicillin-	2154/9/10 12:00	2154/9/12 17:00
13748490	26261739	34808968	Amoxicillin-	2144/6/27 23:00	2144/6/30 11:00
13762454	21447003	38443476	Amoxicillin	2180/1/19 18:00	2180/1/23 01:00
13763521	25821026	32090723	Amoxicillin	2111/1/18 12:00	2111/1/19 11:00
13763521	25821026	32090723	Amoxicillin	2111/1/19 08:00	2111/1/22 09:00
13763521	25821026	32090723	Amoxicillin	2111/1/23 08:00	2111/1/23 15:00
13763521	25821026	32090723	Amoxicillin	2111/1/23 08:00	2111/1/29 17:00
13763521	25821026	32090723	Amoxicillin	2111/2/2 15:00	2111/2/4 17:00
13764208	26437814	35761102	Amoxicillin-	2119/1/11 11:00	2119/1/13 08:00
13764208	26437814	35761102	Amoxicillin-	2119/1/13 11:00	2119/1/17 02:00
13767643	27082271	38023777	Amoxicillin	2152/12/9 18:00	2152/12/10 10:00
13786130	29692487	30348214	Amoxicillin-	2169/7/18 10:00	2169/7/18 11:00
13791232	25434704	33648412	Amoxicillin-	2177/10/20 13:00	2177/10/20 16:00
13791232	25434704	33648412	Amoxicillin-	2177/10/21 11:00	2177/10/23 11:00
13804408	26908606	31508846	Amoxicillin-	2124/4/28 20:00	2124/5/2 06:00
13816151	20053395	38023971	Amoxicillin-	2188/9/2 23:00	2188/9/3 12:00
13823921	24539878	31766669	Amoxicillin	2137/3/25 17:00	2137/3/26 08:00
13837390	26746223	39213250	Amoxicillin-	2171/1/1 11:00	2171/1/2 02:00
13881981	20817928	38092273	Amoxicillin	2153/10/18 10:00	2153/10/18 17:00
13886288	23925448	37808968	Amoxicillin-	2176/11/18 08:00	2176/11/18 14:00
13899653	23821411	31623159	Amoxicillin	2112/4/27 16:00	2112/4/27 18:00
13899653	23821411	31623159	Amoxicillin	2112/4/28 15:00	2112/5/9 16:00
13899653	23821411	31623159	Amoxicillin-	2112/3/31 14:00	2112/4/1 13:00
13899653	23821411	31623159	Amoxicillin-	2112/4/9 12:00	2112/4/10 08:00
13926224	20917385	37509761	Amoxicillin-	2178/5/24 20:00	2178/5/30 18:00
13944280	20642142	36865503	Amoxicillin-	2128/10/23 02:00	2128/10/24 21:00
13965901	24758899	32180681	Amoxicillin-	2181/12/25 14:00	2181/12/28 18:00
13983282	20372482	33807870	Amoxicillin	2193/12/9 13:00	2193/12/9 15:00
13983282	20372482	33807870	Amoxicillin-	2193/12/9 16:00	2193/12/9 23:00
13985166	29770856	37238716	Amoxicillin-	2153/11/19 15:00	2153/11/20 15:00
13985166	29770856	37238716	Amoxicillin-	2153/12/3 00:00	2153/12/3 06:00
13993571	22440139	35722082	Amoxicillin-	2179/8/10 08:00	2179/8/10 19:00
13993861	28782751	37009493	Amoxicillin-	2140/7/27 20:00	2140/7/28 13:00
13993861	28782751	37009493	Amoxicillin-	2140/7/28 20:00	2140/7/28 14:00
13995672	21494992	35831078	Amoxicillin-	2138/6/14 20:00	2138/6/15 15:00
14004638	22463381	36699108	Amoxicillin	2151/2/13 09:00	2151/2/14 07:00
14007589	24920613	37198258	Amoxicillin	2121/4/5 10:00	2121/4/8 22:00
14045654	24160410	31290077	Amoxicillin-	2137/10/18 21:00	2137/10/19 07:00
14055662	26465253	35673045	Amoxicillin-	2173/6/28 20:00	2173/6/29 22:00
14089595	22594270	32316886	Amoxicillin-	2191/4/23 20:00	2191/4/23 20:00
14099520	29117176	37739523	Amoxicillin-	2163/9/12 17:00	2163/9/13 14:00
14111088	27003807	37870234	Amoxicillin	2152/9/18 17:00	2152/9/18 21:00

14132619	24985608	33862764	Amoxicillin-	2119/2/23 13:00	2119/2/28 19:00
14144066	21298790	35439969	Amoxicillin-	2148/4/28 12:00	2148/4/29 02:00
14153931	27597506	30537672	Amoxicillin-	2157/5/18 18:00	2157/5/22 21:00
14159106	22535130	31409242	Amoxicillin-	2175/2/20 13:00	2175/2/24 16:00
14182243	23034067	35804465	Amoxicillin	2171/8/16 10:00	2171/8/16 18:00
14242865	28900801	31717478	Amoxicillin-	2145/8/31 16:00	2145/8/31 15:00
14242865	28900801	31717478	Amoxicillin-	2145/8/31 16:00	2145/9/3 15:00
14243399	23440998	32942857	Amoxicillin-	2166/12/9 09:00	2166/12/9 12:00
14243399	23440998	32942857	Amoxicillin-	2166/12/16 11:00	2166/12/17 18:00
14266489	26077929	38464084	Amoxicillin-	2142/6/5 08:00	2142/6/6 12:00
14272080	20522049	37788909	Amoxicillin	2133/12/25 08:00	2133/12/28 07:00
14272080	20522049	37788909	Amoxicillin-	2133/12/24 20:00	2133/12/25 10:00
14273598	27442726	39698576	Amoxicillin-	2137/4/7 14:00	2137/4/10 19:00
14289094	23247126	30005085	Amoxicillin-	2136/1/24 20:00	2136/1/26 14:00
14295375	27790537	36648673	Amoxicillin	2129/1/7 19:00	2129/1/12 11:00
14316474	21892852	37412118	Amoxicillin-	2148/4/16 10:00	2148/4/17 19:00
14371185	23863622	34462536	Amoxicillin-	2156/6/6 11:00	2156/6/11 11:00
14371185	23863622	34462536	Amoxicillin-	2156/6/11 12:00	2156/6/12 09:00
14371185	23863622	34462536	Amoxicillin-	2156/6/12 10:00	2156/6/13 11:00
14373210	27013158	30997396	Amoxicillin-	2185/6/7 15:00	2185/6/7 21:00
14374577	27718375	32398806	Amoxicillin	2137/1/18 10:00	2137/1/21 19:00
14392547	28668423	39733573	Amoxicillin-	2134/7/3 10:00	2134/7/4 19:00
14399359	28122689	36383457	Amoxicillin-	2143/11/17 14:00	2143/11/17 21:00
14429096	24286687	35774667	Amoxicillin-	2116/1/26 00:00	2116/1/30 01:00
14448498	21770295	39558423	Amoxicillin-	2145/2/25 21:00	2145/3/1 22:00
14459723	20384876	30312039	Amoxicillin	2160/3/10 20:00	2160/3/11 11:00
14459723	20384876	30312039	Amoxicillin	2160/3/11 12:00	2160/3/11 21:00
14459723	20384876	30312039	Amoxicillin	2160/3/12 14:00	2160/3/18 11:00
14477077	29258381	39383761	Amoxicillin-	2166/10/7 19:00	2166/10/14 18:00
14537002	26919590	33173059	Amoxicillin	2169/11/2 09:00	2169/11/2 11:00
14537002	26919590	33173059	Amoxicillin	2169/11/2 21:00	2169/11/2 14:00
14537002	26919590	33173059	Amoxicillin	2169/11/9 09:00	2169/11/12 17:00
14539683	20128314	30941552	Amoxicillin-	2117/1/23 17:00	2117/1/23 18:00
14539683	20128314	30941552	Amoxicillin-	2117/1/23 20:00	2117/1/24 08:00
14539683	20128314	30941552	Amoxicillin-	2117/1/23 20:00	2117/1/27 09:00
14539683	20128314	30941552	Amoxicillin-	2117/1/24 19:00	2117/1/24 04:00
14539683	20128314	30941552	Amoxicillin-	2117/1/27 10:00	2117/1/28 11:00
14539683	20128314	30941552	Amoxicillin-	2117/1/28 12:00	2117/1/29 11:00
14551322	26076652	38772315	Amoxicillin	2143/6/8 10:00	2143/6/13 00:00
14611833	24799971	39767683	Amoxicillin-	2143/1/5 20:00	2143/1/6 20:00
14618211	24100082	32288448	Amoxicillin-	2127/4/20 10:00	2127/4/23 09:00
14622515	27844787	34195512	Amoxicillin-	2142/5/21 11:00	2142/5/21 18:00
14622515	27844787	34195512	Amoxicillin-	2142/5/22 12:00	2142/5/23 08:00
14626007	27226275	35205244	Amoxicillin-	2151/8/1 12:00	2151/8/1 14:00
14626007	27226275	35205244	Amoxicillin-	2151/8/1 12:00	2151/8/1 14:00
14637841	25856326	32841671	Amoxicillin	2126/9/4 10:00	2126/9/4 14:00

14637841	25856326	32841671	Amoxicillin-	2126/9/9 11:00	2126/9/9 10:00
14641622	29999186	32199257	Amoxicillin-	2135/7/15 07:00	2135/7/15 18:00
14646223	21551781	39570721	Amoxicillin-	2170/12/14 10:00	2170/12/14 23:00
14648292	29574391	37115373	Amoxicillin-	2171/2/10 13:00	2171/2/12 20:00
14652380	21737730	39508629	Amoxicillin-	2138/8/29 10:00	2138/8/29 12:00
14662246	28309502	32467158	Amoxicillin-	2202/4/4 20:00	2202/4/7 23:00
14683399	22818662	35577020	Amoxicillin-	2149/2/1 20:00	2149/2/5 22:00
14696096	22350935	35991484	Amoxicillin-	2175/5/30 20:00	2175/6/2 14:00
14759585	20503205	35918049	Amoxicillin-	2164/8/22 12:00	2164/8/26 10:00
14759585	20503205	35918049	Amoxicillin-	2164/8/26 12:00	2164/8/31 19:00
14781720	23795457	39446578	Amoxicillin-	2189/1/21 09:00	2189/1/22 12:00
14805037	24069094	38213456	Amoxicillin-	2113/12/23 12:00	2113/12/23 19:00
14823694	27787169	34319010	Amoxicillin	2118/6/8 21:00	2118/6/9 13:00
14823694	27787169	34319010	Amoxicillin	2118/6/9 14:00	2118/6/16 19:00
14835491	25199819	37374605	Amoxicillin-	2164/11/15 08:00	2164/11/18 15:00
14836889	28634721	34319023	Amoxicillin-	2129/1/10 12:00	2129/1/13 11:00
14839889	29417834	30830382	Amoxicillin-	2127/11/11 14:00	2127/11/12 09:00
14856688	21718734	31479025	Amoxicillin	2180/9/8 10:00	2180/9/9 20:00
14876689	24123487	39624002	Amoxicillin-	2194/11/14 08:00	2194/11/16 01:00
14879689	21235518	35810727	Amoxicillin-	2117/7/6 03:00	2117/7/6 18:00
14936164	20185704	32448624	Amoxicillin-	2162/9/4 19:00	2162/9/8 21:00
14949139	23259506	35131321	Amoxicillin	2145/11/29 18:00	2145/11/30 10:00
14993789	22586480	39721629	Amoxicillin-	2136/10/30 08:00	2136/10/30 09:00
15015081	21669554	37587186	Amoxicillin-	2159/2/4 15:00	2159/2/4 16:00
15021508	21471846	31474741	Amoxicillin-	2152/4/23 13:00	2152/4/27 22:00
15041265	27532773	36055576	Amoxicillin-	2165/3/29 07:00	2165/3/30 17:00
15050647	27925109	30235174	Amoxicillin-	2126/3/1 07:00	2126/3/6 18:00
15050647	27925109	30235174	Amoxicillin-	2126/3/1 19:00	2126/3/1 20:00
15055839	25660999	38853975	Amoxicillin-	2137/7/26 20:00	2137/7/27 09:00
15055839	25660999	38853975	Amoxicillin-	2137/7/29 14:00	2137/8/3 00:00
15055839	25660999	38853975	Amoxicillin-	2137/8/3 14:00	2137/8/4 01:00
15055839	25660999	38853975	Amoxicillin-	2137/8/4 00:00	2137/8/4 10:00
15055839	25660999	38853975	Amoxicillin-	2137/8/4 14:00	2137/8/5 10:00
15055839	25660999	38853975	Amoxicillin-	2137/8/5 10:00	2137/8/6 12:00
15055839	25660999	38853975	Amoxicillin-	2137/8/5 18:00	2137/8/5 18:00
15081288	24836583	38506734	Amoxicillin-	2130/3/17 17:00	2130/3/20 09:00
15081288	24836583	38506734	Amoxicillin-	2130/3/20 08:00	2130/3/21 20:00
15102074	21503329	38075215	Amoxicillin-	2163/2/18 09:00	2163/2/19 15:00
15102074	21503329	38075215	Amoxicillin-	2163/2/23 08:00	2163/2/28 23:00
15107144	20138568	35964351	Amoxicillin	2120/1/3 02:00	2120/1/4 04:00
15107144	20138568	35964351	Amoxicillin	2120/1/3 14:00	2120/1/3 14:00
15119590	26430459	38553297	Amoxicillin	2118/7/5 14:00	2118/7/8 23:00
15123526	28239166	33110536	Amoxicillin	2145/9/17 12:00	2145/9/17 18:00
15126168	25130370	35847409	Amoxicillin	2144/2/10 13:00	2144/2/15 20:00
15147932	21065624	33408756	Amoxicillin-	2168/8/14 12:00	2168/8/17 19:00
15178141	21202073	34146910	Amoxicillin	2158/3/1 09:00	2158/3/2 19:00

15179110	27994691	30336793	Amoxicillin-	2124/3/4 07:00	2124/3/4 14:00
15180326	20480350	39836146	Amoxicillin-	2158/1/2 21:00	2158/1/3 17:00
15198477	23021484	33189945	Amoxicillin-	2151/5/26 12:00	2151/5/26 16:00
15198477	23021484	33189945	Amoxicillin-	2151/5/26 17:00	2151/5/27 21:00
15209343	23114711	35902394	Amoxicillin-	2120/6/20 09:00	2120/6/20 20:00
15209343	23114711	35902394	Amoxicillin-	2120/6/20 21:00	2120/6/23 19:00
15222924	28908204	33306272	Amoxicillin-	2153/9/12 12:00	2153/9/14 17:00
15240836	25822110	33911427	Amoxicillin-	2182/8/26 12:00	2182/8/31 11:00
15250661	21529005	31625877	Amoxicillin-	2162/10/19 19:00	2162/10/20 07:00
15280135	23185373	36671240	Amoxicillin	2187/10/29 23:00	2187/11/1 21:00
15309481	21312439	34856309	Amoxicillin	2151/11/16 17:00	2151/11/17 11:00
15309481	21312439	34856309	Amoxicillin	2151/11/17 12:00	2151/11/18 11:00
15357247	20546766	30662121	Amoxicillin-	2136/11/16 11:00	2136/11/17 14:00
15357247	20546766	30662121	Amoxicillin-	2136/11/17 12:00	2136/11/18 11:00
15357247	20546766	30662121	Amoxicillin-	2136/11/14 14:00	2136/11/14 21:00
15370732	22935332	32798179	Amoxicillin-	2179/10/25 20:00	2179/10/26 08:00
15377080	27143313	33165226	Amoxicillin-	2183/11/25 09:00	2183/11/25 19:00
15379156	20063548	36646574	Amoxicillin-	2175/3/23 14:00	2175/3/24 23:00
15387461	22918339	31745657	Amoxicillin-	2149/7/14 21:00	2149/7/15 09:00
15387461	22918339	31745657	Amoxicillin-	2149/7/15 21:00	2149/7/20 08:00
15423614	24817985	31737615	Amoxicillin-	2162/11/11 15:00	2162/11/17 11:00
15437636	28028740	39956136	Amoxicillin-	2165/10/8 09:00	2165/10/15 16:00
15463196	28650948	39357775	Amoxicillin-	2157/6/7 20:00	2157/6/8 10:00
15463196	28650948	39357775	Amoxicillin-	2157/6/8 20:00	2157/6/9 20:00
15479218	28411600	38486717	Amoxicillin	2167/10/15 11:00	2167/10/27 11:00
15479218	28411600	38486717	Amoxicillin	2167/10/27 12:00	2167/10/30 11:00
15510891	24221160	37262080	Amoxicillin	2166/9/21 16:00	2166/9/22 16:00
15510891	24221160	37262080	Amoxicillin	2166/9/24 16:00	2166/9/25 21:00
15511352	26248412	31898214	Amoxicillin-	2178/7/3 09:00	2178/7/4 17:00
15511352	26248412	31898214	Amoxicillin-	2178/7/4 21:00	2178/7/5 19:00
15515193	25955360	38193820	Amoxicillin-	2156/7/3 14:00	2156/7/6 00:00
15516903	26455292	39609533	Amoxicillin-	2179/6/17 20:00	2179/6/18 19:00
15549491	25301773	31758850	Amoxicillin-	2156/10/4 03:00	2156/10/9 20:00
15551558	26228316	36132348	Amoxicillin-	2157/1/28 08:00	2157/1/28 17:00
15554091	22146346	38642672	Amoxicillin-	2134/3/30 14:00	2134/3/31 15:00
15562994	25638541	30373977	Amoxicillin-	2135/2/5 11:00	2135/2/5 23:00
15564969	27532535	35263980	Amoxicillin	2176/10/13 18:00	2176/10/14 19:00
15566609	26543049	30058490	Amoxicillin-	2123/3/24 19:00	2123/4/4 11:00
15578020	23331845	38229559	Amoxicillin-	2161/2/3 10:00	2161/2/3 19:00
15585879	24388156	33905596	Amoxicillin	2128/6/21 16:00	2128/6/25 20:00
15610823	26657269	36053507	Amoxicillin-	2167/9/27 18:00	2167/9/27 17:00
15610823	26657269	36053507	Amoxicillin-	2167/9/27 18:00	2167/9/29 21:00
15613449	20213419	34442018	Amoxicillin-	2138/7/17 15:00	2138/7/18 20:00
15618038	21390834	30036517	Amoxicillin-	2168/10/4 13:00	2168/10/9 20:00
15650925	24438854	39523345	Amoxicillin-	2184/6/23 11:00	2184/6/26 20:00
15682194	24569744	39936606	Amoxicillin-	2145/6/2 16:00	2145/6/6 23:00

15682194	24569744	39936606	Amoxicillin-	2145/6/7 04:00	2145/6/17 17:00
15690806	23321044	32123185	Amoxicillin-	2124/8/5 11:00	2124/8/5 13:00
15690806	23321044	32123185	Amoxicillin-	2124/8/5 23:00	2124/8/11 11:00
15716202	21531668	33919649	Amoxicillin	2160/10/22 03:00	2160/10/22 09:00
15716281	23583286	36323204	Amoxicillin-	2121/4/22 16:00	2121/4/27 13:00
15731682	24603823	36772522	Amoxicillin-	2144/9/19 12:00	2144/9/19 11:00
15731682	24603823	36772522	Amoxicillin-	2144/9/19 12:00	2144/9/20 18:00
15747460	20331438	39526480	Amoxicillin-	2135/4/17 14:00	2135/4/19 23:00
15776550	26078996	35323229	Amoxicillin-	2117/3/9 14:00	2117/3/13 13:00
15777308	29150364	30873498	Amoxicillin-	2112/8/12 12:00	2112/8/13 23:00
15783228	20017191	39430975	Amoxicillin-	2132/12/4 17:00	2132/12/5 09:00
15798883	25610841	33753547	Amoxicillin-	2110/3/24 20:00	2110/3/25 18:00
15810023	29958361	38369185	Amoxicillin	2182/9/29 20:00	2182/9/29 11:00
15874204	29660139	39934716	Amoxicillin-	2151/11/11 11:00	2151/11/11 21:00
15897499	23580227	37759666	Amoxicillin	2114/11/24 17:00	2114/11/25 17:00
15913148	22690587	31197768	Amoxicillin-	2166/3/22 08:00	2166/3/22 19:00
15918842	25980684	37078411	Amoxicillin	2116/6/29 14:00	2116/6/29 15:00
15923856	20994916	34897322	Amoxicillin-	2134/8/24 11:00	2134/8/24 11:00
15941722	28673719	31319568	Amoxicillin-	2129/4/27 10:00	2129/4/28 09:00
15941722	28673719	31319568	Amoxicillin-	2129/4/28 10:00	2129/4/28 20:00
15942634	22682133	35889304	Amoxicillin	2116/1/6 12:00	2116/1/6 21:00
15952165	21530208	35549689	Amoxicillin-	2175/2/1 11:00	2175/2/2 20:00
15995260	24512660	39730719	Amoxicillin-	2124/11/25 17:00	2124/11/27 23:00
15996793	24931995	32091966	Amoxicillin-	2139/6/12 18:00	2139/6/13 09:00
16022256	27633132	31153217	Amoxicillin-	2118/12/6 20:00	2118/12/6 16:00
16060022	23139342	31951680	Amoxicillin-	2156/2/29 10:00	2156/3/1 18:00
16103717	24551770	32348213	Amoxicillin-	2141/11/16 11:00	2141/11/20 16:00
16118301	20048854	38513783	Amoxicillin-	2167/10/13 11:00	2167/10/15 18:00
16127252	27322379	37502590	Amoxicillin-	2170/12/16 17:00	2170/12/18 21:00
16144348	23848836	37603493	Amoxicillin-	2147/6/3 12:00	2147/6/4 17:00
16205152	28727183	30534026	Amoxicillin-	2130/5/24 18:00	2130/5/28 12:00
16209336	21988238	37003779	Amoxicillin-	2146/9/15 12:00	2146/9/17 10:00
16221385	26259520	33525664	Amoxicillin	2181/11/27 19:00	2181/11/28 13:00
16223641	26330409	35258308	Amoxicillin-	2114/10/5 12:00	2114/10/11 09:00
16224237	27373667	32436950	Amoxicillin-	2170/6/18 20:00	2170/6/19 22:00
16227072	24617470	36043532	Amoxicillin	2164/11/30 00:00	2164/12/4 11:00
16236685	22361608	32719055	Amoxicillin-	2145/11/22 09:00	2145/11/22 19:00
16250782	28595599	39519845	Amoxicillin-	2114/5/13 12:00	2114/5/13 18:00
16268194	23300738	35397581	Amoxicillin	2173/6/25 16:00	2173/6/26 21:00
16268194	23300738	35397581	Amoxicillin	2173/6/26 04:00	2173/6/28 17:00
16271079	23479444	39290548	Amoxicillin	2169/5/7 18:00	2169/5/8 19:00
16271251	20536660	39241853	Amoxicillin-	2182/4/6 12:00	2182/4/6 22:00
16271251	20536660	39241853	Amoxicillin-	2182/4/6 10:00	2182/4/6 11:00
16275349	20401398	35610412	Amoxicillin-	2139/8/16 14:00	2139/8/16 21:00
16275349	20401398	35610412	Amoxicillin-	2139/8/16 14:00	2139/8/16 21:00
16280495	23168926	38712394	Amoxicillin-	2164/1/14 13:00	2164/1/15 17:00

16285950	22978966	37190362	Amoxicillin-	2199/8/10 20:00	2199/8/15 09:00
16306611	26974016	37507054	Amoxicillin-	2181/6/25 20:00	2181/6/25 16:00
16306611	26974016	37507054	Amoxicillin-	2181/6/25 20:00	2181/6/25 19:00
16306611	26974016	37507054	Amoxicillin-	2181/6/26 20:00	2181/6/28 16:00
16313615	29966121	32164707	Amoxicillin-	2149/9/13 18:00	2149/9/14 11:00
16313615	29966121	32164707	Amoxicillin-	2149/9/14 19:00	2149/9/15 12:00
16326056	20121937	32375386	Amoxicillin-	2123/9/28 16:00	2123/10/3 09:00
16327683	27906486	38293696	Amoxicillin-	2129/4/9 08:00	2129/4/9 08:00
16327683	27906486	38293696	Amoxicillin-	2129/4/10 02:00	2129/4/14 01:00
16334176	27172835	37945394	Amoxicillin	2124/10/8 15:00	2124/10/8 21:00
16335352	26731114	36030785	Amoxicillin	2191/4/24 19:00	2191/4/28 23:00
16354891	25271240	33414220	Amoxicillin	2172/4/14 17:00	2172/4/15 15:00
16356099	29331285	32742724	Amoxicillin-	2123/10/23 20:00	2123/10/23 19:00
16356118	28866315	34780406	Amoxicillin-	2180/8/13 12:00	2180/8/13 18:00
16364130	27580078	37352309	Amoxicillin	2129/12/12 16:00	2129/12/15 18:00
16397275	23393160	35146789	Amoxicillin-	2133/12/31 14:00	2134/1/2 22:00
16403658	27527450	37254591	Amoxicillin-	2172/7/9 09:00	2172/7/12 18:00
16407305	27627597	38593284	Amoxicillin-	2133/1/24 20:00	2133/1/29 22:00
16413192	23579377	31202577	Amoxicillin-	2144/7/27 22:00	2144/7/30 13:00
16420761	22348316	30279037	Amoxicillin-	2168/9/22 10:00	2168/9/23 13:00
16424633	26508758	34124448	Amoxicillin-	2116/8/26 15:00	2116/9/8 01:00
16440032	22351089	31357123	Amoxicillin-	2146/6/27 16:00	2146/6/30 18:00
16456443	25411209	30447105	Amoxicillin-	2188/9/28 11:00	2188/9/28 12:00
16472043	27670949	38838793	Amoxicillin-	2145/8/9 08:00	2145/8/13 18:00
16473192	27668896	32359651	Amoxicillin-	2163/5/29 16:00	2163/5/30 10:00
16473192	27668896	32359651	Amoxicillin-	2163/5/30 16:00	2163/5/30 18:00
16483436	20116402	37510531	Amoxicillin-	2176/3/18 18:00	2176/3/24 07:00
16483436	20116402	37510531	Amoxicillin-	2176/3/24 10:00	2176/3/26 09:00
16489093	27878812	38573250	Amoxicillin-	2112/6/12 11:00	2112/6/13 07:00
16496279	29176711	35424694	Amoxicillin-	2167/1/29 20:00	2167/2/1 13:00
16540581	21878647	33407760	Amoxicillin-	2150/3/25 09:00	2150/3/26 08:00
16540581	21878647	33407760	Amoxicillin-	2150/3/26 08:00	2150/3/26 22:00
16567910	26423982	36188727	Amoxicillin-	2128/12/21 19:00	2128/12/23 22:00
16592058	22007481	33485591	Amoxicillin-	2139/10/4 12:00	2139/10/10 11:00
16610463	24249306	33204925	Amoxicillin	2154/6/16 18:00	2154/6/17 12:00
16617539	20619236	34892678	Amoxicillin-	2110/3/26 09:00	2110/3/28 20:00
16667797	29736092	34051051	Amoxicillin-	2174/11/21 17:00	2174/11/22 20:00
16679373	27683797	30149950	Amoxicillin-	2123/3/7 20:00	2123/3/7 18:00
16679373	27683797	30149950	Amoxicillin-	2123/3/7 20:00	2123/3/10 22:00
16691318	23568585	34018437	Amoxicillin-	2180/3/12 19:00	2180/3/13 10:00
16691318	23568585	34018437	Amoxicillin-	2180/3/13 19:00	2180/3/14 14:00
16711075	22347991	30191018	Amoxicillin-	2123/5/29 17:00	2123/6/1 10:00
16749523	20715585	35426484	Amoxicillin-	2138/5/12 22:00	2138/5/13 19:00
16750404	25310445	34479285	Amoxicillin-	2171/4/23 12:00	2171/5/2 01:00
16790159	20556058	38001807	Amoxicillin-	2127/6/4 12:00	2127/6/4 18:00
16811048	22494786	39281008	Amoxicillin-	2171/4/19 07:00	2171/4/21 16:00

16819157	29207053	32070868	Amoxicillin-	2116/6/24 20:00	2116/6/26 23:00
16839166	21283565	30901980	Amoxicillin	2126/3/22 14:00	2126/3/23 19:00
16857974	20257174	38434716	Amoxicillin-	2128/11/13 16:00	2128/11/14 12:00
16859143	25895643	30076359	Amoxicillin-	2125/6/19 00:00	2125/6/22 19:00
16873164	23710972	31330227	Amoxicillin	2140/3/18 15:00	2140/3/18 14:00
16873164	23710972	31330227	Amoxicillin	2140/3/18 15:00	2140/3/20 23:00
16887555	28143695	35716501	Amoxicillin-	2153/1/1 09:00	2153/1/1 10:00
16887555	28143695	35716501	Amoxicillin-	2153/1/1 11:00	2153/1/2 08:00
16896235	20855320	36851727	Amoxicillin-	2165/6/5 08:00	2165/6/6 00:00
16897269	21223469	35866792	Amoxicillin-	2163/4/26 10:00	2163/4/26 11:00
16905121	25141736	32960510	Amoxicillin-	2111/4/15 11:00	2111/4/15 11:00
16905121	25141736	32960510	Amoxicillin-	2111/4/15 23:00	2111/4/20 10:00
16905121	25141736	32960510	Amoxicillin-	2111/4/20 11:00	2111/4/20 20:00
16922206	22719584	34047954	Amoxicillin	2148/4/26 16:00	2148/4/26 15:00
16922206	22719584	34047954	Amoxicillin	2148/4/26 16:00	2148/4/26 21:00
16922206	22719584	34047954	Amoxicillin-	2148/4/26 22:00	2148/4/28 13:00
16925602	26327093	36418222	Amoxicillin	2182/11/9 13:00	2182/11/13 08:00
16925602	26327093	36418222	Amoxicillin	2182/11/13 08:00	2182/11/13 08:00
16925602	26327093	36418222	Amoxicillin	2182/11/13 08:00	2182/11/15 19:00
16925602	26327093	36418222	AMOXicillin	2182/11/9 10:00	2182/11/9 12:00
16928727	27784279	32079161	Amoxicillin-	2152/3/4 20:00	2152/3/4 19:00
16928727	27784279	32079161	Amoxicillin-	2152/3/7 20:00	2152/3/8 19:00
16937336	27057825	38958113	Amoxicillin	2146/3/9 19:00	2146/3/10 10:00
16946907	26681158	35154803	Amoxicillin-	2142/7/7 20:00	2142/7/16 19:00
16967171	29835251	37185043	Amoxicillin-	2151/8/2 02:00	2151/8/1 21:00
16976998	20034762	32324096	Amoxicillin	2164/6/9 01:00	2164/6/16 20:00
16979986	29542651	39944977	Amoxicillin-	2159/10/10 12:00	2159/10/13 01:00
17001450	20445016	38756705	Amoxicillin-	2143/10/5 11:00	2143/10/5 22:00
17014029	21628869	38842975	Amoxicillin-	2127/8/26 12:00	2127/8/27 08:00
17047957	23361485	38141354	Amoxicillin-	2125/3/2 18:00	2125/3/4 18:00
17051865	20392650	36094197	Amoxicillin	2120/9/20 12:00	2120/9/21 06:00
17056572	29937835	34027544	Amoxicillin	2192/5/24 10:00	2192/5/25 13:00
17056572	29937835	34027544	Amoxicillin	2192/5/26 08:00	2192/5/31 10:00
17056572	29937835	34027544	Amoxicillin	2192/5/31 20:00	2192/5/31 21:00
17076625	22563409	36582269	Amoxicillin-	2135/2/17 20:00	2135/2/18 12:00
17076625	22563409	36582269	Amoxicillin-	2135/2/19 14:00	2135/2/23 21:00
17078498	22161926	37731040	Amoxicillin-	2189/11/6 17:00	2189/11/6 19:00
17123238	26729329	32160176	Amoxicillin-	2157/7/28 10:00	2157/7/28 14:00
17123538	26328341	34870097	Amoxicillin-	2180/3/28 15:00	2180/4/2 14:00
17155697	27527916	36719412	Amoxicillin	2165/1/11 18:00	2165/1/12 10:00
17155701	21781012	38398288	Amoxicillin-	2128/4/20 15:00	2128/4/26 22:00
17158562	29949077	38522183	Amoxicillin-	2123/4/15 01:00	2123/4/16 00:00
17158955	26717744	32752508	Amoxicillin-	2146/6/15 21:00	2146/6/16 07:00
17160548	26470937	30481344	Amoxicillin-	2145/11/21 09:00	2145/11/24 21:00
17173309	22443690	34735850	Amoxicillin-	2149/3/19 11:00	2149/3/19 17:00
17173309	22443690	34735850	Amoxicillin-	2149/3/19 16:00	2149/3/19 20:00

17173309	22443690	34735850	Amoxicillin-	2149/3/20 00:00	2149/3/24 23:00
17189198	28924169	31473734	Amoxicillin-	2140/7/27 15:00	2140/7/27 20:00
17191974	26927498	35517021	Amoxicillin-	2129/6/17 14:00	2129/6/22 21:00
17202838	29504891	34389377	Amoxicillin-	2147/4/13 15:00	2147/4/18 14:00
17204052	28903397	37361121	Amoxicillin-	2178/10/24 15:00	2178/10/24 21:00
17219425	28362481	32856939	Amoxicillin-	2150/9/24 12:00	2150/9/28 23:00
17219425	28362481	32856939	Amoxicillin-	2150/9/29 00:00	2150/9/29 23:00
17220323	25700666	30004242	Amoxicillin	2180/11/19 07:00	2180/11/19 18:00
17220323	25700666	30004242	Amoxicillin	2180/11/20 12:00	2180/11/21 19:00
17229758	26591890	31497434	Amoxicillin	2141/8/20 13:00	2141/8/20 22:00
17246136	24604333	37637080	Amoxicillin-	2128/5/8 09:00	2128/5/9 13:00
17246136	24604333	37637080	Amoxicillin-	2128/5/5 10:00	2128/5/8 08:00
17251257	27045507	38384732	Amoxicillin-	2131/8/17 11:00	2131/8/19 09:00
17260849	20160410	33084408	Amoxicillin-	2157/3/11 15:00	2157/3/12 16:00
17260849	20160410	33084408	Amoxicillin-	2157/3/12 22:00	2157/3/12 22:00
17260849	20160410	33084408	Amoxicillin-	2157/3/13 00:00	2157/3/16 21:00
17276069	27635465	31695091	Amoxicillin-	2119/10/28 12:00	2119/10/29 22:00
17292877	21129597	39921028	Amoxicillin-	2190/2/1 14:00	2190/2/2 05:00
17292877	21129597	39921028	Amoxicillin-	2190/2/2 06:00	2190/2/3 16:00
17292877	21129597	39921028	Amoxicillin-	2190/2/6 09:00	2190/2/6 21:00
17297876	24303829	39620847	Amoxicillin-	2118/2/7 15:00	2118/2/8 07:00
17297876	24303829	39620847	Amoxicillin-	2118/2/8 15:00	2118/2/9 14:00
17341852	28503284	36121759	Amoxicillin-	2177/1/5 09:00	2177/1/5 18:00
17375855	24072797	39477599	Amoxicillin-	2137/4/16 10:00	2137/4/17 21:00
17380809	25207758	36856170	Amoxicillin-	2188/8/10 10:00	2188/8/10 11:00
17380809	25207758	36856170	Amoxicillin-	2188/8/10 22:00	2188/8/10 18:00
17393871	28717667	31590722	Amoxicillin-	2159/12/8 16:00	2159/12/10 12:00
17395049	29880166	33387861	Amoxicillin-	2172/12/17 15:00	2172/12/17 20:00
17408369	28968531	35231303	Amoxicillin-	2136/10/30 11:00	2136/10/30 10:00
17421003	22811508	36342317	Amoxicillin-	2189/3/21 14:00	2189/3/22 18:00
17427308	20564752	31268481	Amoxicillin-	2155/9/29 16:00	2155/10/4 11:00
17440103	29881676	37689746	Amoxicillin-	2188/6/15 18:00	2188/6/16 23:00
17447280	28523367	38296093	Amoxicillin	2196/4/12 09:00	2196/4/17 21:00
17461434	28378325	30748856	Amoxicillin	2126/5/30 19:00	2126/6/1 18:00
17482549	23975230	31731813	Amoxicillin-	2172/9/26 08:00	2172/10/4 18:00
17509177	26528363	32672640	Amoxicillin	2159/9/2 08:00	2159/9/4 21:00
17509177	26528363	32672640	Amoxicillin-	2159/9/2 07:00	2159/9/2 07:00
17512630	26719728	39598788	Amoxicillin-	2157/3/16 11:00	2157/3/18 18:00
17514642	22144247	39236072	Amoxicillin-	2142/2/4 20:00	2142/2/6 18:00
17533998	22139413	37598308	Amoxicillin	2121/1/6 14:00	2121/1/8 11:00
17570754	21316847	37626263	Amoxicillin	2118/5/19 18:00	2118/5/20 16:00
17571919	21908176	39734844	Amoxicillin-	2171/6/26 10:00	2171/6/26 17:00
17584398	22082288	36504972	Amoxicillin	2149/12/10 12:00	2149/12/13 23:00
17602938	21922268	30232259	Amoxicillin	2154/12/10 17:00	2154/12/10 23:00
17611233	20214316	37415746	Amoxicillin-	2137/11/26 11:00	2137/11/27 20:00
17637743	27510539	36937821	Amoxicillin-	2131/10/16 12:00	2131/10/16 21:00

17645263	26165406	38217045	Amoxicillin-	2126/6/6 08:00	2126/6/7 11:00
17656727	22432603	33130940	Amoxicillin-	2138/11/15 13:00	2138/11/16 09:00
17680528	22962546	38833700	Amoxicillin	2113/5/2 20:00	2113/5/16 19:00
17683498	21856524	39141359	Amoxicillin-	2152/4/6 08:00	2152/4/11 09:00
17741310	23903316	31231621	Amoxicillin-	2140/8/20 14:00	2140/8/20 22:00
17760392	27407944	33905519	Amoxicillin	2119/4/28 09:00	2119/4/29 12:00
17824252	21839853	31387148	Amoxicillin-	2151/5/10 20:00	2151/5/12 22:00
17830851	24111560	32558836	Amoxicillin-	2119/4/10 20:00	2119/4/11 20:00
17838494	22190208	39857839	Amoxicillin-	2113/11/2 17:00	2113/11/3 05:00
17858057	22407099	31194864	Amoxicillin-	2138/11/17 10:00	2138/11/17 16:00
17868867	27579357	35907382	Amoxicillin-	2153/1/4 15:00	2153/1/6 18:00
17885100	23392333	31289726	Amoxicillin-	2121/7/5 18:00	2121/7/6 23:00
17908001	21375043	34852935	Amoxicillin-	2136/7/27 19:00	2136/7/29 23:00
17916774	20491610	35467275	Amoxicillin-	2165/9/6 10:00	2165/9/6 10:00
17916774	20491610	35467275	Amoxicillin-	2165/9/6 22:00	2165/9/12 02:00
17929966	26303906	31278863	Amoxicillin-	2155/12/6 00:00	2155/12/8 20:00
17958577	26573186	30251251	Amoxicillin-	2126/1/11 08:00	2126/1/13 21:00
17958577	26573186	30251251	Amoxicillin-	2126/1/13 20:00	2126/1/15 19:00
17981773	26448854	34638264	Amoxicillin	2145/7/1 22:00	2145/7/3 10:00
17982020	29831232	38686261	Amoxicillin-	2128/11/6 15:00	2128/11/7 23:00
17989583	21126877	35813455	Amoxicillin-	2149/10/5 11:00	2149/10/7 21:00
17994170	21243868	31442095	Amoxicillin-	2159/7/23 15:00	2159/7/23 21:00
18019825	21387908	35289144	Amoxicillin-	2157/11/28 13:00	2157/11/30 12:00
18023644	24924655	31528797	Amoxicillin	2123/12/3 12:00	2123/12/4 11:00
18024998	24567210	38247671	Amoxicillin-	2184/6/8 10:00	2184/6/10 12:00
18024998	24567210	38247671	Amoxicillin-	2184/6/10 18:00	2184/6/11 08:00
18030687	23100477	30625864	Amoxicillin	2184/6/13 14:00	2184/6/13 20:00
18036384	25963110	36870399	Amoxicillin-	2142/3/26 16:00	2142/3/27 08:00
18046498	21154892	30103416	Amoxicillin-	2180/4/15 12:00	2180/4/15 11:00
18046498	21154892	30103416	Amoxicillin-	2180/4/15 12:00	2180/4/16 14:00
18070061	20327114	38657278	Amoxicillin-	2121/6/22 15:00	2121/6/22 16:00
18070061	20327114	38657278	Amoxicillin-	2121/6/22 17:00	2121/6/23 21:00
18077825	28556984	34701203	Amoxicillin-	2144/7/29 21:00	2144/8/3 20:00
18079495	21595876	31378707	Amoxicillin-	2204/5/27 14:00	2204/5/30 21:00
18079519	26824537	30526491	Amoxicillin-	2172/5/23 18:00	2172/5/28 10:00
18081075	20584998	36611218	Amoxicillin-	2142/3/13 10:00	2142/3/16 23:00
18081075	20584998	36611218	Amoxicillin-	2142/3/13 19:00	2142/3/13 19:00
18088944	22787187	35913928	Amoxicillin	2148/3/24 18:00	2148/3/28 13:00
18100032	22404782	33250702	Amoxicillin-	2115/11/2 10:00	2115/11/4 16:00
18132013	23086340	37086726	Amoxicillin	2176/6/12 07:00	2176/6/12 11:00
18135870	25369826	31847662	Amoxicillin-	2127/10/15 11:00	2127/10/16 19:00
18137539	21957886	37481477	Amoxicillin-	2142/10/1 15:00	2142/10/2 12:00
18141227	28884412	36872097	Amoxicillin	2123/1/2 16:00	2123/1/3 13:00
18141227	28884412	36872097	Amoxicillin	2123/1/3 14:00	2123/1/3 19:00
18143098	21736175	32058979	Amoxicillin-	2170/5/26 11:00	2170/5/27 18:00
18144491	21377327	34064983	Amoxicillin-	2184/6/3 23:00	2184/6/5 15:00

18145609	23225134	32703142	Amoxicillin-	2185/2/28 07:00	2185/2/28 10:00
18159987	27933922	34035036	Amoxicillin	2149/9/20 19:00	2149/9/24 10:00
18190098	20394032	36959615	Amoxicillin-	2145/4/14 13:00	2145/4/15 20:00
18205763	20732919	38047603	Amoxicillin-	2143/11/1 08:00	2143/11/1 07:00
18208298	21399659	31643820	Amoxicillin-	2123/5/9 09:00	2123/5/9 17:00
18219834	26310730	39076422	Amoxicillin	2168/12/26 22:00	2168/12/26 22:00
18219834	26310730	39076422	Amoxicillin	2168/12/26 22:00	2168/12/27 20:00
18219834	26310730	39076422	Amoxicillin	2168/12/26 22:00	2168/12/28 05:00
18219834	26310730	39076422	Amoxicillin	2168/12/28 08:00	2169/1/2 12:00
18219834	26310730	39076422	Amoxicillin	2169/1/2 20:00	2169/1/4 22:00
18228802	23692539	31506424	Amoxicillin-	2113/2/26 12:00	2113/2/27 20:00
18238983	23550863	36950541	Amoxicillin	2149/8/23 18:00	2149/8/24 22:00
18260162	27718396	38623475	Amoxicillin-	2143/10/16 14:00	2143/10/16 18:00
18279430	28843066	35530775	Amoxicillin-	2155/11/30 03:00	2155/11/30 08:00
18295542	29083442	39784741	Amoxicillin	2157/12/2 16:00	2157/12/2 16:00
18318959	29036812	38854340	Amoxicillin-	2117/6/15 14:00	2117/6/15 14:00
18318959	29036812	38854340	Amoxicillin-	2117/6/15 15:00	2117/6/17 21:00
18322075	21664116	31999918	Amoxicillin-	2142/4/25 12:00	2142/4/25 16:00
18322555	22290182	31605135	Amoxicillin	2141/5/14 13:00	2141/5/15 04:00
18329823	20601126	34663319	Amoxicillin	2179/10/30 20:00	2179/10/30 11:00
18334297	23790893	38573028	Amoxicillin-	2186/12/21 20:00	2186/12/25 17:00
18335638	22889129	39241284	Amoxicillin	2180/8/21 20:00	2180/8/22 22:00
18343701	26846317	30139247	Amoxicillin-	2158/7/2 21:00	2158/7/5 17:00
18409091	25810474	36373933	Amoxicillin-	2188/1/28 00:00	2188/1/30 09:00
18437407	26552728	37722446	Amoxicillin-	2179/9/1 16:00	2179/9/8 02:00
18437407	26552728	37722446	Amoxicillin-	2179/9/11 02:00	2179/9/17 20:00
18437407	26552728	37722446	Amoxicillin-	2179/9/17 20:00	2179/9/18 16:00
18441942	26731051	30874552	Amoxicillin-	2134/3/9 11:00	2134/3/13 09:00
18441942	26731051	30874552	Amoxicillin-	2134/3/13 11:00	2134/3/13 15:00
18443789	20437552	37762932	Amoxicillin	2174/10/3 19:00	2174/10/3 19:00
18444540	26809380	35154260	Amoxicillin-	2156/12/6 18:00	2156/12/7 23:00
18447676	25359272	33600295	Amoxicillin-	2140/4/3 10:00	2140/4/3 11:00
18447676	25359272	33600295	Amoxicillin-	2140/4/3 22:00	2140/4/4 20:00
18447676	25359272	33600295	Amoxicillin-	2140/4/4 14:00	2140/4/4 20:00
18455165	22765209	34503775	AMOXicillin	2184/7/17 08:00	2184/7/22 09:00
18495040	22436392	35501216	Amoxicillin-	2167/9/15 21:00	2167/9/16 20:00
18499026	27880403	30039798	Amoxicillin-	2137/9/23 16:00	2137/9/24 21:00
18530734	27298586	36654885	Amoxicillin	2160/2/2 15:00	2160/2/3 20:00
18536618	26558632	30752639	Amoxicillin-	2169/4/7 18:00	2169/4/16 10:00
18536618	26558632	30752639	Amoxicillin-	2169/4/20 11:00	2169/4/22 09:00
18539613	21213705	32293219	Amoxicillin-	2132/9/21 11:00	2132/9/21 14:00
18542020	21044579	39414369	Amoxicillin-	2175/8/20 20:00	2175/8/21 15:00
18542020	21044579	39414369	Amoxicillin-	2175/8/21 20:00	2175/8/26 22:00
18558087	21205497	31876097	Amoxicillin-	2179/7/25 20:00	2179/7/25 17:00
18558763	20714880	37208356	Amoxicillin	2121/2/9 15:00	2121/2/14 22:00
18558946	23481036	32468504	Amoxicillin-	2112/6/2 20:00	2112/6/2 17:00

18559699	24834668	36262115	Amoxicillin-	2124/12/8 14:00	2124/12/9 21:00
18560132	29479073	30242774	Amoxicillin-	2204/8/31 09:00	2204/9/6 08:00
18581793	22289488	36941749	Amoxicillin-	2145/12/6 13:00	2145/12/8 00:00
18618974	20909981	33776852	Amoxicillin-	2133/3/4 11:00	2133/3/5 02:00
18637496	26725335	30173242	Amoxicillin-	2167/7/5 12:00	2167/7/8 09:00
18655887	27294406	39076846	Amoxicillin-	2142/5/17 08:00	2142/5/17 09:00
18655887	27294406	39076846	Amoxicillin-	2142/5/17 16:00	2142/5/17 10:00
18655887	27294406	39076846	Amoxicillin-	2142/5/17 16:00	2142/5/19 18:00
18687658	26913211	34769822	Amoxicillin-	2125/6/28 13:00	2125/6/29 21:00
18694693	28597346	39591720	Amoxicillin-	2123/3/26 20:00	2123/3/27 18:00
18737673	29105226	32070739	Amoxicillin-	2133/1/6 16:00	2133/1/6 15:00
18742839	21822700	34602174	Amoxicillin-	2187/7/3 17:00	2187/7/5 20:00
18769342	29336424	38238834	Amoxicillin-	2183/1/19 20:00	2183/1/26 19:00
18769680	23250071	35847112	Amoxicillin-	2138/3/4 11:00	2138/3/4 21:00
18771560	20395830	32391848	Amoxicillin-	2131/9/4 20:00	2131/9/4 11:00
18771560	20395830	32391848	Amoxicillin-	2131/9/4 20:00	2131/9/5 22:00
18773543	21334216	33488244	Amoxicillin	2126/8/4 17:00	2126/8/6 08:00
18809506	25963140	30242598	Amoxicillin-	2148/9/10 21:00	2148/9/11 08:00
18809506	25963140	30242598	Amoxicillin-	2148/9/11 09:00	2148/9/12 00:00
18860097	26748189	37872238	Amoxicillin-	2133/9/22 15:00	2133/9/26 11:00
18886241	22554453	37461538	Amoxicillin-	2141/3/14 18:00	2141/3/15 21:00
18903466	24332484	31765253	Amoxicillin-	2197/7/15 12:00	2197/7/15 19:00
18908042	20381307	39944889	Amoxicillin	2179/3/6 00:00	2179/3/6 19:00
18920063	26252331	34361157	Amoxicillin-	2140/6/28 12:00	2140/7/1 14:00
18920063	26252331	34361157	Amoxicillin-	2140/7/1 15:00	2140/7/4 09:00
18920063	26252331	34361157	Amoxicillin-	2140/7/4 15:00	2140/7/5 14:00
18944827	25780665	34813660	Amoxicillin	2173/9/13 00:00	2173/9/13 18:00
18998265	26102340	33503828	Amoxicillin	2153/8/5 22:00	2153/8/7 11:00
18998265	26102340	33503828	Amoxicillin	2153/8/7 22:00	2153/8/8 04:00
19059275	21125311	31676937	Amoxicillin-	2148/8/5 09:00	2148/8/9 00:00
19089771	25222525	34424454	Amoxicillin-	2167/3/6 13:00	2167/3/8 18:00
19097890	23075303	36764379	Amoxicillin	2196/9/15 18:00	2196/9/16 18:00
19100245	24526280	30841557	Amoxicillin-	2171/1/8 20:00	2171/1/10 00:00
19109196	24189635	38935660	Amoxicillin	2174/10/9 23:00	2174/10/12 09:00
19109196	24189635	38935660	Amoxicillin	2174/10/11 18:00	2174/10/14 20:00
19123301	20795597	38637695	Amoxicillin-	2136/5/29 15:00	2136/5/30 19:00
19129667	20863601	31812799	Amoxicillin-	2165/1/9 18:00	2165/1/9 20:00
19129667	20863601	31812799	Amoxicillin-	2165/1/9 20:00	2165/1/10 20:00
19130440	27258229	39799866	Amoxicillin-	2172/4/27 14:00	2172/4/27 19:00
19147314	28850468	31402227	Amoxicillin-	2120/1/29 00:00	2120/1/30 19:00
19147900	23485171	31986193	Amoxicillin-	2121/5/10 09:00	2121/5/11 22:00
19165656	29527014	39133485	Amoxicillin-	2135/6/11 17:00	2135/6/14 09:00
19165656	29527014	39133485	Amoxicillin-	2135/6/14 20:00	2135/6/15 19:00
19203810	22191809	38890956	Amoxicillin	2160/6/2 19:00	2160/6/3 09:00
19203810	22191809	38890956	Amoxicillin	2160/6/3 10:00	2160/6/8 22:00
19214634	24843141	32609921	Amoxicillin	2135/11/21 22:00	2135/11/23 16:00

19226833	20895515	30049503	Amoxicillin	2133/2/22 11:00	2133/2/22 13:00
19226833	20895515	30049503	Amoxicillin-	2133/2/22 18:00	2133/2/23 11:00
19226833	20895515	30049503	Amoxicillin-	2133/2/22 20:00	2133/2/22 17:00
19226833	20895515	30049503	Amoxicillin-	2133/2/23 18:00	2133/2/24 12:00
19226833	20895515	30049503	Amoxicillin-	2133/2/24 18:00	2133/3/5 09:00
19239525	20236920	35955058	Amoxicillin	2189/1/24 09:00	2189/1/27 08:00
19241989	27097138	39195328	Amoxicillin-	2167/5/2 14:00	2167/5/2 17:00
19287914	27288489	31368984	Amoxicillin-	2110/7/14 09:00	2110/7/14 09:00
19287914	27288489	31368984	Amoxicillin-	2110/7/16 15:00	2110/7/19 09:00
19291648	24167341	35962834	Amoxicillin-	2125/10/6 10:00	2125/10/6 13:00
19291648	24167341	35962834	Amoxicillin-	2125/10/6 14:00	2125/10/7 21:00
19295124	24117507	39323941	Amoxicillin	2187/1/20 21:00	2187/1/21 15:00
19295124	24117507	39323941	Amoxicillin-	2187/1/22 11:00	2187/1/24 23:00
19318463	27215532	39084108	Amoxicillin-	2196/8/4 15:00	2196/8/5 20:00
19328746	21841188	33236024	Amoxicillin	2154/6/28 15:00	2154/6/29 17:00
19351106	23027929	30187303	Amoxicillin	2130/8/21 09:00	2130/8/21 18:00
19365784	25478150	37643192	AMOXicillin	2196/3/12 19:00	2196/3/16 17:00
19378928	25081327	32222688	Amoxicillin-	2150/10/16 21:00	2150/10/17 23:00
19385005	26677308	37387855	Amoxicillin-	2154/12/24 10:00	2154/12/25 07:00
19418928	20248299	37455406	Amoxicillin-	2112/11/16 21:00	2112/11/17 12:00
19423967	24812246	30750724	Amoxicillin	2177/7/1 14:00	2177/7/7 19:00
19423967	24812246	30750724	Amoxicillin	2177/7/7 20:00	2177/7/9 10:00
19426292	22824095	32950082	Amoxicillin	2156/7/31 15:00	2156/8/1 01:00
19443634	28839059	34971107	Amoxicillin-	2180/9/9 12:00	2180/9/9 17:00
19443634	28839059	34971107	Amoxicillin-	2180/9/9 20:00	2180/9/10 19:00
19448629	29219112	34852120	Amoxicillin-	2160/10/25 09:00	2160/10/25 17:00
19456890	24126123	34840865	Amoxicillin	2188/7/5 14:00	2188/7/8 00:00
19457252	23692825	33581256	Amoxicillin-	2178/2/23 09:00	2178/2/24 08:00
19457252	23692825	33581256	Amoxicillin-	2178/2/24 09:00	2178/2/25 23:00
19464920	28303858	37883377	Amoxicillin-	2160/12/29 15:00	2160/12/31 16:00
19472874	22955354	34694944	Amoxicillin-	2168/2/27 01:00	2168/2/27 00:00
19472874	22955354	34694944	Amoxicillin-	2168/2/27 01:00	2168/2/27 08:00
19475346	20047275	37522159	Amoxicillin	2116/10/29 11:00	2116/10/29 10:00
19475346	20047275	37522159	Amoxicillin	2116/10/29 11:00	2116/10/30 10:00
19500641	23824657	39800204	Amoxicillin	2144/2/23 13:00	2144/3/1 18:00
19524729	20877271	37959301	Amoxicillin-	2130/4/26 11:00	2130/4/27 16:00
19524729	20877271	37959301	Amoxicillin-	2130/4/29 10:00	2130/4/29 18:00
19524873	25104323	30460078	Amoxicillin-	2110/9/4 10:00	2110/9/5 01:00
19529371	27332988	31833050	Amoxicillin-	2166/10/31 14:00	2166/11/1 22:00
19540374	20284410	38421631	Amoxicillin-	2151/1/16 13:00	2151/1/21 10:00
19591207	24652930	32439226	Amoxicillin-	2126/11/4 10:00	2126/11/4 10:00
19598941	27198141	32167782	Amoxicillin-	2190/5/3 10:00	2190/5/5 21:00
19624301	28505006	39545590	Amoxicillin-	2119/11/22 15:00	2119/11/23 10:00
19624301	28505006	39545590	Amoxicillin-	2119/11/23 15:00	2119/11/25 16:00
19650793	20792056	34592853	Amoxicillin-	2160/2/12 12:00	2160/2/20 18:00
19693912	28103782	34762448	Amoxicillin-	2146/9/4 07:00	2146/9/4 10:00

19725417	27668527	37524474	Amoxicillin-	2167/9/4 19:00	2167/9/8 23:00
19728718	28730433	36446276	Amoxicillin-	2148/1/22 09:00	2148/1/26 14:00
19728718	28730433	36446276	Amoxicillin-	2148/1/28 09:00	2148/1/29 18:00
19728718	28730433	36446276	Amoxicillin-	2148/1/30 01:00	2148/2/1 20:00
19731685	21005127	35025499	Amoxicillin	2166/1/25 18:00	2166/1/26 12:00
19731685	21005127	35025499	Amoxicillin	2166/1/27 07:00	2166/2/3 10:00
19731685	21005127	35025499	Amoxicillin	2166/2/5 10:00	2166/2/6 14:00
19731685	21005127	35025499	Amoxicillin	2166/2/6 15:00	2166/2/10 21:00
19734799	28834851	32797128	Amoxicillin	2178/8/10 12:00	2178/8/10 17:00
19806212	25284876	36539073	Amoxicillin-	2133/6/28 16:00	2133/7/1 20:00
19821197	21574277	39975569	Amoxicillin-	2122/9/18 15:00	2122/9/19 21:00
19832355	22483349	36331629	Amoxicillin-	2161/5/27 18:00	2161/5/27 18:00
19840468	23322937	33669892	Amoxicillin-	2133/2/17 15:00	2133/2/18 20:00
19848285	22505521	31487845	Amoxicillin-	2122/4/5 10:00	2122/4/5 20:00
19867017	24776727	33976214	Amoxicillin-	2124/4/19 12:00	2124/4/19 18:00
19867017	24776727	33976214	Amoxicillin-	2124/4/19 20:00	2124/4/21 16:00
19869590	24842662	34062535	Amoxicillin-	2117/2/3 14:00	2117/2/6 23:00
19882264	26291550	33567531	Amoxicillin-	2184/3/30 20:00	2184/3/31 21:00
19903197	26747168	35247961	Amoxicillin-	2195/4/11 13:00	2195/4/12 13:00
19927184	24340154	30261124	Amoxicillin-	2145/2/15 16:00	2145/2/15 23:00
19936782	28486132	30150327	Amoxicillin-	2154/2/11 13:00	2154/2/12 06:00
19936782	28486132	30150327	Amoxicillin-	2154/2/12 08:00	2154/2/12 13:00
19955909	29894050	34000757	Amoxicillin-	2180/7/31 11:00	2180/7/31 18:00
19958337	29604470	30159100	Amoxicillin	2152/9/29 19:00	2152/10/1 19:00
19992312	25379817	30412504	Amoxicillin-	2158/6/30 17:00	2158/7/1 09:00
19992312	25379817	30412504	Amoxicillin-	2158/7/1 17:00	2158/7/1 20:00

dose_val_rx	doses_per_2	route	dose_unit_r	form_unit_d	dose_total
875	2	PO/NG	mg	TAB	1750
875	2	PO/NG	mg	TAB	1750
875	2	PO/NG	mg	TAB	1750
500	3	PO/NG	mg	CAP	1500
500	3	PO/NG	mg	CAP	1500
500	3	PO/NG	mg	CAP	1500
500	1	PO/NG	mg	CAP	500
500	2	PO	mg	SYR	1000
500	2	PO	mg	CAP	1000
500	2	PO	mg	CAP	1000
500	3	PO	mg	TAB	1500
500	3	PO	mg	TAB	1500
500	2	PO/NG	mg	TAB	1000
875	2	PO/NG	mg	TAB	1750
875	2	PO/NG	mg	TAB	1750
500	3	PO/NG	mg	TAB	1500
500	1	PO/NG	mg	TAB	500
875	2	PO/NG	mg	TAB	1750
500	3	PO/NG	mg	SYR	1500
500	3	PO/NG	mg	CAP	1500
2000	1	PO/NG	mg	CAP	2000
875	2	PO/NG	mg	TAB	1750
875	2	PO/NG	mg	TAB	1750
500	3	PO	mg	SYR	1500
875	2	PO/NG	mg	TAB	1750
875	2	PO/NG	mg	TAB	1750
875	2	PO/NG	mg	TAB	1750
875	2	PO/NG	mg	TAB	1750
500	1	PO/NG	mg	CAP	500
500	2	PO	mg	CAP	1000
875	2	PO/NG	mg	TAB	1750
1000	2	PO/NG	mg	SYR	2000
875	2	PO/NG	mg	TAB	1750
1000	2	PO/NG	mg	CAP	2000
875	2	PO	mg	TAB	1750
500	1	PO/NG	mg	TAB	500
500	1	PO/NG	mg	TAB	500
875	2	PO/NG	mg	TAB	1750
875	2	PO/NG	mg	TAB	1750
875	1	PO/NG	mg	TAB	875
875	2	PO/NG	mg	TAB	1750
875	2	PO/NG	mg	TAB	1750
500	3	PO/NG	mg	TAB	1500
500	2	PO/NG	mg	CAP	1000
875	2	PO/NG	mg	TAB	1750

500	2	PO/NG	mg	TAB	1000
500	2	PO/NG	mg	TAB	1000
875	2	PO/NG	mg	TAB	1750
875	2	PO/NG	mg	TAB	1750
875	2	PO/NG	mg	TAB	1750
875	2	PO/NG	mg	TAB	1750
1000	2	PO	mg	CAP	2000
875	2	PO/NG	mg	TAB	1750
875	2	PO/NG	mg	TAB	1750
875	2	PO/NG	mg	TAB	1750
1000	2	PO/NG	mg	mL	2000
1000	2	PO/NG	mg	CAP	2000
875	2	PO/NG	mg	TAB	1750
500	1	PO/NG	mg	CAP	500
1000	2	PO	mg	CAP	2000
875	2	PO/NG	mg	TAB	1750
875	2	PO/NG	mg	TAB	1750
500	3	PO/NG	mg	TAB	1500
500	2	PO/NG	mg	TAB	1000
500	2	PO/NG	mg	TAB	1000
875	2	PO/NG	mg	TAB	1750
1000	2	NG	mg	SYR	2000
500	3	PO/NG	mg	CAP	1500
875	2	PO/NG	mg	TAB	1750
875	2	PO/NG	mg	TAB	1750
875	2	PO/NG	mg	TAB	1750
500	3	PO/NG	mg	SYR	1500
875	2	PO/NG	mg	TAB	1750
1000	2	PO/NG	mg	CAP	2000
1000	2	PO/NG	mg	SYR	2000
875	2	PO/NG	mg	TAB	1750
875	2	PO/NG	mg	TAB	1750
500	2	PO/NG	mg	CAP	1000
500	2	PO/NG	mg	CAP	1000
500	2	PO/NG	mg	CAP	1000
500	2	PO	mg	CAP	1000
500	2	PO	mg	CAP	1000
875	2	PO	mg	TAB	1750
875	2	PO	mg	TAB	1750
500	3	PO	mg	TAB	1500
875	2	PO/NG	mg	TAB	1750
875	2	PO/NG	mg	TAB	1750
500	1	PO	mg	CAP	500
500	2	PO/NG	mg	TAB	1000
500	3	PO/NG	mg	SYR	1500
875	2	PO/NG	mg	TAB	1750

500	2	PO	mg	CAP	1000
875	2	PO/NG	mg	TAB	1750
875	2	PO/NG	mg	TAB	1750
500	2	PO/NG	mg	TAB	1000
500	3	PO	mg	TAB	1500
500	2	PO/NG	mg	SYR	1000
500	2	PO/NG	mg	CAP	1000
875	2	PO/NG	mg	TAB	1750
875	2	PO/NG	mg	TAB	1750
500	2	PO/NG	mg	TAB	1000
500	2	PO/NG	mg	TAB	1000
500	2	PO/NG	mg	TAB	1000
875	2	PO/NG	mg	TAB	1750
500	2	PO	mg	CAP	1000
875	2	PO/NG	mg	TAB	1750
500	2	PO/NG	mg	TAB	1000
500	1	PO/NG	mg	TAB	500
500	1	PO	mg	TAB	500
500	1	PO/NG	mg	CAP	500
1000	2	PO/NG	mg	CAP	2000
1000	2	PO/NG	mg	SYR	2000
500	1	PO	mg	CAP	500
500	1	PO	mg	SYR	500
500	2	PO	mg	CAP	1000
500	2	PO	mg	CAP	1000
500	2	PO	mg	SYR	1000
500	2	PO/NG	mg	SYR	1000
500	2	PO/NG	mg	TAB	1000
500	3	PO/NG	mg	TAB	1500
500	3	PO/NG	mg	CAP	1500
875	2	PO/NG	mg	TAB	1750
875	2	PO/NG	mg	TAB	1750
500	2	PO/NG	mg	TAB	1000
500	2	PO/NG	mg	TAB	1000
500	2	PO	mg	CAP	1000
500	3	PO/NG	mg	CAP	1500
500	2	PO/NG	mg	CAP	1000
500	1	PO/NG	mg	CAP	500
875	2	PO/NG	mg	TAB	1750
500	4	PO/NG	mg	CAP	2000
500	4	PO/NG	mg	CAP	2000
1000	2	PO/NG	mg	SYR	2000
875	2	PO/NG	mg	TAB	1750
500	3	PO/NG	mg	TAB	1500
500	4	PO	mg	CAP	2000
500	4	PO	mg	CAP	2000

500	2	PO/NG	mg	TAB	1000
875	2	PO/NG	mg	TAB	1750
875	2	PO/NG	mg	TAB	1750
875	2	PO/NG	mg	TAB	1750
875	2	PO/NG	mg	TAB	1750
875	2	PO/NG	mg	TAB	1750
875	2	PO/NG	mg	TAB	1750
875	2	PO/NG	mg	TAB	1750
500	3	PO/NG	mg	CAP	1500
500	3	PO/NG	mg	CAP	1500
875	2	PO/NG	mg	TAB	1750
500	3	PO/NG	mg	CAP	1500
875	2	PO/NG	mg	TAB	1750
875	2	PO/NG	mg	TAB	1750
875	2	PO/NG	mg	TAB	1750
875	2	PO	mg	TAB	1750
875	2	PO	mg	TAB	1750
875	2	PO	mg	TAB	1750
500	3	PO/NG	mg	SYR	1500
500	3	PO/NG	mg	SYR	1500
875	2	PO/NG	mg	TAB	1750
500	3	PO/NG	mg	SYR	1500
500	3	PO	mg	TAB	1500
1000	3	PO	mg	CAP	3000
875	2	PO	mg	TAB	1750
875	2	PO/NG	mg	TAB	1750
500	2	PO/NG	mg	TAB	1000
875	3	PO	mg	TAB	2625
500	3	PO/NG	mg	TAB	1500
500	3	PO/NG	mg	TAB	1500
500	3	PO/NG	mg	TAB	1500
500	2	PO/NG	mg	SYR	1000
500	2	PO/NG	mg	SYR	1000
500	2	PO/NG	mg	SYR	1000
875	2	PO/NG	mg	TAB	1750
500	3	PO/NG	mg	TAB	1500
875	2	PO/NG	mg	TAB	1750
875	2	PO	mg	TAB	1750
500	1	PO/NG	mg	TAB	500
500	1	PO/NG	mg	TAB	500
500	1	PO/NG	mg	TAB	500
500	1	PO/NG	mg	TAB	500
500	2	PO	mg	TAB	1000
875	2	PO/NG	mg	TAB	1750
875	2	PO/NG	mg	TAB	1750
500	3	PO/NG	mg	SYR	1500

875	2	PO/NG	mg	TAB	1750
500	3	PO/NG	mg	CAP	1500
875	2	PO/NG	mg	TAB	1750
500	2	PO/NG	mg	TAB	1000
500	2	PO/NG	mg	SYR	1000
500	2	PO/NG	mg	SYR	1000
500	2	PO/NG	mg	TAB	1000
500	1	PO	mg	CAP	500
500	1	PO	mg	TAB	500
500	1	PO	mg	TAB	500
500	1	PO	mg	TAB	500
1000	2	PO/NG	mg	CAP	2000
1000	2	PO/NG	mg	SYR	2000
1000	2	PO/NG	mg	SYR	2000
500	3	PO/NG	mg	TAB	1500
500	3	PO/NG	mg	TAB	1500
875	2	PO/NG	mg	SYR	1750
875	2	PO/NG	mg	TAB	1750
500	2	PO/NG	mg	SYR	1000
500	2	PO/NG	mg	CAP	1000
500	3	PO	mg	TAB	1500
875	2	PO	mg	TAB	1750
875	2	PO/NG	mg	TAB	1750
875	2	PO/NG	mg	TAB	1750
500	3	PO/NG	mg	SYR	1500
500	1	PO/NG	mg	TAB	500
875	2	PO/NG	mg	TAB	1750
875	2	PO/NG	mg	TAB	1750
875	2	PO/NG	mg	TAB	1750
500	3	PO/NG	mg	TAB	1500
875	2	PO/NG	mg	TAB	1750
875	2	PO	mg	TAB	1750
875	2	PO/NG	mg	TAB	1750
875	2	PO/NG	mg	TAB	1750
875	2	PO/NG	mg	TAB	1750
500	3	PO/NG	mg	CAP	1500
500	2	PO/NG	mg	TAB	1000
500	3	PO/NG	mg	CAP	1500
500	2	PO/NG	mg	CAP	1000
500	3	PO	mg	TAB	1500
875	2	PO/NG	mg	TAB	1750
875	2	PO	mg	TAB	1750
500	3	PO	mg	TAB	1500
500	2	PO/NG	mg	TAB	1000
500	2	PO/NG	mg	TAB	1000
500	2	PO/NG	mg	TAB	1000

1000	2	PO/NG	mg	CAP	2000
1000	2	PO/NG	mg	mL	2000
500	3	PO/NG	mg	CAP	1500
875	2	PO	mg	TAB	1750
250	3	PO	mg	CAP	750
500	3	PO/NG	mg	SYR	1500
500	3	PO/NG	mg	TAB	1500
500	3	PO/NG	mg	CAP	1500
500	3	PO/NG	mg	CAP	1500
500	3	PO/NG	mg	SYR	1500
875	2	PO/NG	mg	SYR	1750
875	2	PO/NG	mg	TAB	1750
500	3	PO	mg	TAB	1500
500	3	PO/NG	mg	CAP	1500
500	2	PO/NG	mg	TAB	1000
1000	2	PO/NG	mg	CAP	2000
875	2	PO/NG	mg	SYR	1750
875	2	PO/NG	mg	SYR	1750
875	2	PO/NG	mg	TAB	1750
500	3	PO/NG	mg	TAB	1500
500	3	PO/NG	mg	CAP	1500
500	3	PO/NG	mg	SYR	1500
500	3	PO/NG	mg	TAB	1500
500	3	PO/NG	mg	TAB	1500
875	2	PO	mg	TAB	1750
1000	2	PO/NG	mg	CAP	2000
500	2	PO/NG	mg	CAP	1000
500	2	PO	mg	CAP	1000
500	3	PO	mg	CAP	1500
500	3	PO/NG	mg	SYR	1500
875	2	PO/NG	mg	TAB	1750
500	2	PO	mg	CAP	1000
500	2	PO	mg	CAP	1000
875	2	PO/NG	mg	TAB	1750
500	2	PO/NG	mg	CAP	1000
500	3	PO	mg	TAB	1500
500	3	PO/NG	mg	TAB	1500
500	2	PO	mg	CAP	1000
500	3	PO/NG	mg	SYR	1500
500	3	PO	mg	CAP	1500
875	2	PO/NG	mg	TAB	1750
500	3	PO/NG	mg	TAB	1500
500	2	PO/NG	mg	TAB	1000
875	2	PO/NG	mg	TAB	1750
875	2	PO/NG	mg	TAB	1750
500	2	PO	mg	TAB	1000

500	2	PO/NG	mg	TAB	1000
1000	2	PO/NG	mg	CAP	2000
500	2	PO	mg	CAP	1000
875	3	PO/NG	mg	TAB	2625
875	2	PO	mg	TAB	1750
500	3	PO/NG	mg	TAB	1500
1000	2	PO/NG	mg	CAP	2000
500	3	PO	mg	TAB	1500
875	2	PO/NG	mg	TAB	1750
875	2	PO/NG	mg	TAB	1750
500	1	PO/NG	mg	CAP	500
500	1	PO/NG	mg	SYR	500
500	2	PO	mg	TAB	1000
500	3	PO	mg	TAB	1500
875	2	PO	mg	TAB	1750
875	2	PO/NG	mg	TAB	1750
2000	1	PO/NG	mg	CAP	2000
875	2	PO/NG	mg	TAB	1750
875	2	PO/NG	mg	TAB	1750
875	2	PO/NG	mg	TAB	1750
875	2	PO/NG	mg	TAB	1750
500	1	PO	mg	CAP	500
500	1	PO	mg	CAP	500
500	3	PO/NG	mg	SYR	1500
875	2	PO/NG	mg	TAB	1750
500	2	PO	mg	TAB	1000
500	2	PO	mg	TAB	1000
875	2	PO/NG	mg	TAB	1750
875	2	PO/NG	mg	TAB	1750
500	3	PO/NG	mg	SYR	1500
875	2	PO/NG	mg	TAB	1750
875	2	PO/NG	mg	TAB	1750
500	3	PO/NG	mg	TAB	1500
500	2	PO/NG	mg	TAB	1000
500	3	PO/NG	mg	CAP	1500
500	2	PO	mg	TAB	1000
500	2	PO/NG	mg	TAB	1000
1000	2	PO/NG	mg	CAP	2000
875	2	PO/NG	mg	TAB	1750
500	2	PO	mg	TAB	1000
500	2	PO/NG	mg	TAB	1000
875	2	PO/NG	mg	TAB	1750
875	2	PO/NG	mg	TAB	1750
875	2	PO/NG	mg	TAB	1750
875	2	PO/NG	mg	TAB	1750
875	2	PO/NG	mg	SYR	1750

875	2	PO/NG	mg	SYR	1750
875	2	PO/NG	mg	TAB	1750
875	2	PO/NG	mg	TAB	1750
875	2	PO/NG	mg	TAB	1750
875	2	PO/NG	mg	TAB	1750
875	1	PO/NG	mg	TAB	875
500	3	PO/NG	mg	CAP	1500
500	3	PO/NG	mg	CAP	1500
500	2	PO	mg	TAB	1000
500	2	PO	mg	TAB	1000
500	2	PO	mg	TAB	1000
875	2	PO/NG	mg	TAB	1750
875	2	PO/NG	mg	TAB	1750
500	3	PO/NG	mg	SYR	1500
875	2	PO/NG	mg	TAB	1750
500	3	PO/NG	mg	TAB	1500
875	2	PO/NG	mg	TAB	1750
500	3	PO/NG	mg	TAB	1500
875	2	PO	mg	TAB	1750
875	2	PO/NG	mg	TAB	1750
500	2	PO/NG	mg	TAB	1000
875	2	PO	mg	TAB	1750
500	2	PO	mg	TAB	1000
500	3	PO/NG	mg	TAB	1500
500	2	PO/NG	mg	TAB	1000
875	2	PO/NG	mg	SYR	1750
875	2	PO/NG	mg	TAB	1750
1000	2	PO/NG	mg	mL	2000
500	3	PO/NG	mg	SYR	1500
500	2	PO/NG	mg	SYR	1000
875	2	PO/NG	mg	TAB	1750
500	2	PO/NG	mg	TAB	1000
875	2	PO/NG	mg	TAB	1750
1000	3	PO/NG	mg	CAP	3000
875	2	PO/NG	mg	TAB	1750
500	3	PO	mg	TAB	1500
875	2	PO/NG	mg	TAB	1750
875	2	PO/NG	mg	TAB	1750
875	2	PO/NG	mg	TAB	1750
500	2	PO/NG	mg	TAB	1000
875	2	PO/NG	mg	TAB	1750
500	2	PO/NG	mg	TAB	1000
500	2	PO/NG	mg	CAP	1000
500	3	PO	mg	CAP	1500
500	2	PO/NG	mg	TAB	1000
875	2	PO/NG	mg	TAB	1750

875	2	PO/NG	mg	TAB	1750
1000	2	PO/NG	mg	CAP	2000
875	2	PO/NG	mg	TAB	1750
500	3	PO/NG	mg	TAB	1500
875	2	PO/NG	mg	TAB	1750
875	2	PO/NG	mg	TAB	1750
500	2	PO	mg	CAP	1000
500	2	PO/NG	mg	CAP	1000
500	1	PO/NG	mg	CAP	500
500	1	PO/NG	mg	mL	500
500	1	PO/NG	mg	CAP	500
500	2	PO/NG	mg	CAP	1000
875	2	PO/NG	mg	TAB	1750
875	2	PO/NG	mg	TAB	1750
500	2	PO/NG	mg	CAP	1000
875	2	PO/NG	mg	TAB	1750
875	2	PO/NG	mg	TAB	1750
875	2	PO/NG	mg	TAB	1750
500	1	PO/NG	mg	TAB	500
500	2	PO/NG	mg	TAB	1000
500	3	PO/NG	mg	CAP	1500
500	2	PO/NG	mg	SYR	1000
500	3	PO	mg	CAP	1500
875	2	PO/NG	mg	TAB	1750
500	3	PO/NG	mg	CAP	1500
500	3	PO/NG	mg	CAP	1500
875	1	PO/NG	mg	TAB	875
875	1	PO/NG	mg	TAB	875
875	2	PO/NG	mg	TAB	1750
500	2	PO/NG	mg	TAB	1000
875	2	PO/NG	mg	TAB	1750
500	3	PO/NG	mg	CAP	1500
875	2	PO/NG	mg	TAB	1750
500	3	PO/NG	mg	TAB	1500
500	2	PO/NG	mg	TAB	1000
500	2	PO/NG	mg	TAB	1000
875	2	PO/NG	mg	TAB	1750
875	2	PO/NG	mg	TAB	1750
875	2	PO/NG	mg	TAB	1750
1000	2	PO	mg	CAP	2000
500	3	PO	mg	CAP	1500
875	2	PO/NG	mg	TAB	1750
875	2	PO/NG	mg	TAB	1750
875	2	PO/NG	mg	TAB	1750
500	1	PO/NG	mg	TAB	500
500	3	PO/NG	mg	CAP	1500

875	1	PO/NG	mg	TAB	875
875	2	PO/NG	mg	TAB	1750
875	2	PO/NG	mg	TAB	1750
875	2	PO/NG	mg	TAB	1750
500	3	PO/NG	mg	CAP	1500
875	2	PO/NG	mg	TAB	1750
875	2	PO/NG	mg	TAB	1750
875	2	PO	mg	TAB	1750
875	2	PO/NG	mg	TAB	1750
875	2	PO/NG	mg	TAB	1750
500	2	PO/NG	mg	CAP	1000
500	2	PO/NG	mg	TAB	1000
875	2	PO/NG	mg	TAB	1750
500	3	PO/NG	mg	TAB	1500
500	2	PO/NG	mg	CAP	1000
500	1	PO/NG	mg	TAB	500
875	2	PO/NG	mg	TAB	1750
875	2	PO/NG	mg	SYR	1750
875	2	PO/NG	mg	TAB	1750
875	2	PO	mg	TAB	1750
500	1	PO/NG	mg	CAP	500
875	2	PO/NG	mg	TAB	1750
500	3	PO	mg	TAB	1500
500	3	PO/NG	mg	TAB	1500
875	2	PO/NG	mg	TAB	1750
500	2	PO/NG	mg	CAP	1000
1000	2	PO/NG	mg	CAP	2000
500	1	PO/NG	mg	CAP	500
875	2	PO/NG	mg	TAB	1750
1000	2	PO	mg	CAP	2000
1000	2	PO	mg	CAP	2000
500	3	PO/NG	mg	SYR	1500
500	3	PO/NG	mg	SYR	1500
500	3	PO/NG	mg	SYR	1500
500	2	PO/NG	mg	SYR	1000
500	3	PO/NG	mg	SYR	1500
500	1	PO/NG	mg	SYR	500
500	1	PO/NG	mg	SYR	500
500	1	PO	mg	CAP	500
875	2	PO/NG	mg	TAB	1750
500	1	PO/NG	mg	TAB	500
875	3	PO	mg	TAB	2625
875	3	PO	mg	TAB	2625
500	2	PO/NG	mg	SYR	1000
500	2	PO/NG	mg	TAB	1000
1000	2	PO/NG	mg	CAP	2000

875	2	PO/NG	mg	TAB	1750
500	3	PO/NG	mg	TAB	1500
500	3	PO	mg	TAB	1500
875	2	PO/NG	mg	TAB	1750
875	2	PO/NG	mg	SYR	1750
875	2	PO/NG	mg	TAB	1750
500	2	PO/NG	mg	TAB	1000
500	3	PO/NG	mg	TAB	1500
500	3	PO	mg	TAB	1500
500	3	PO	mg	TAB	1500
500	2	PO/NG	mg	TAB	1000
875	2	PO	mg	TAB	1750
500	3	PO/NG	mg	CAP	1500
500	3	PO/NG	mg	CAP	1500
500	2	PO/NG	mg	SYR	1000
500	3	PO/NG	mg	TAB	1500
500	2	PO/NG	mg	TAB	1000
500	3	PO/NG	mg	CAP	1500
875	2	PO/NG	mg	TAB	1750
250	3	PO/NG	mg	TAB	750
500	2	PO/NG	mg	TAB	1000
500	3	PO/NG	mg	CAP	1500
875	2	PO/NG	mg	TAB	1750
875	2	PO/NG	mg	TAB	1750
875	2	PO/NG	mg	TAB	1750
500	2	PO/NG	mg	TAB	1000
875	2	PO/NG	mg	SYR	1750
875	2	PO/NG	mg	TAB	1750
500	2	PO/NG	mg	SYR	1000
500	2	PO/NG	mg	SYR	1000
500	3	PO	mg	TAB	1500
500	3	PO	mg	SYR	1500
500	3	PO	mg	SYR	1500
500	3	PO/NG	mg	TAB	1500
250	3	PO/NG	mg	TAB	750
500	2	PO/NG	mg	TAB	1000
500	2	PO/NG	mg	TAB	1000
875	2	PO/NG	mg	TAB	1750
875	2	PO/NG	mg	TAB	1750
500	2	PO	mg	CAP	1000
500	2	PO	mg	CAP	1000
500	3	PO/NG	mg	CAP	1500
1000	2	PO	mg	CAP	2000
500	1	PO	mg	CAP	500
875	2	PO	mg	TAB	1750
500	3	PO/NG	mg	CAP	1500

875	1	PO/NG	mg	TAB	875
875	2	PO/NG	mg	TAB	1750
500	2	PO/NG	mg	SYR	1000
500	2	PO/NG	mg	SYR	1000
875	2	PO	mg	TAB	1750
875	2	PO	mg	SYR	1750
500	2	PO/NG	mg	TAB	1000
500	2	PO/NG	mg	TAB	1000
500	2	PO/NG	mg	TAB	1000
1000	2	PO/NG	mg	CAP	2000
500	2	PO/NG	mg	CAP	1000
500	2	PO/NG	mg	SYR	1000
500	3	PO/NG	mg	SYR	1500
500	3	PO/NG	mg	SYR	1500
875	2	PO/NG	mg	TAB	1750
875	2	PO/NG	mg	TAB	1750
875	2	PO/NG	mg	TAB	1750
500	2	PO/NG	mg	TAB	1000
875	2	PO/NG	mg	TAB	1750
500	2	PO/NG	mg	TAB	1000
875	2	PO/NG	mg	TAB	1750
500	3	PO/NG	mg	SYR	1500
875	2	PO/NG	mg	TAB	1750
875	2	PO/NG	mg	SYR	1750
1000	2	PO/NG	mg	SYR	2000
1000	2	PO/NG	mg	SYR	2000
500	2	PO	mg	CAP	1000
500	2	PO	mg	CAP	1000
875	2	PO/NG	mg	TAB	1750
875	2	PO/NG	mg	TAB	1750
875	2	PO/NG	mg	TAB	1750
500	3	PO/NG	mg	TAB	1500
875	2	PO/NG	mg	TAB	1750
875	2	PO/NG	mg	TAB	1750
875	2	PO/NG	mg	TAB	1750
500	1	PO/NG	mg	TAB	500
500	3	PO	mg	CAP	1500
500	3	PO/NG	mg	SYR	1500
500	3	PO	mg	TAB	1500
500	3	PO/NG	mg	CAP	1500
500	2	PO/NG	mg	TAB	1000
875	2	PO/NG	mg	TAB	1750
875	2	PO/NG	mg	TAB	1750
875	2	PO/NG	mg	TAB	1750
875	2	PO/NG	mg	TAB	1750
875	2	PO/NG	mg	TAB	1750

500	2	PO/NG	mg	TAB	1000
500	2	PO/NG	mg	TAB	1000
500	2	PO/NG	mg	TAB	1000
500	3	PO/NG	mg	CAP	1500
500	2	PO	mg	TAB	1000
875	2	PO	mg	TAB	1750
875	2	PO	mg	TAB	1750
875	2	PO/NG	mg	TAB	1750
875	2	PO/NG	mg	TAB	1750
500	1	PO/NG	mg	TAB	500
875	2	PO/NG	mg	TAB	1750
875	2	PO	mg	TAB	1750
500	3	PO/NG	mg	CAP	1500
875	2	PO/NG	mg	TAB	1750
500	2	PO/NG	mg	CAP	1000
875	2	PO	mg	TAB	1750
1000	2	PO/NG	mg	CAP	2000
500	3	PO	mg	TAB	1500
875	2	PO/NG	mg	TAB	1750
875	2	PO/NG	mg	TAB	1750
1000	2	PO/NG	mg	CAP	2000
500	3	PO/NG	mg	TAB	1500
500	2	PO/NG	mg	TAB	1000
500	3	PO/NG	mg	TAB	1500
875	2	PO	mg	TAB	1750
875	2	PO	mg	TAB	1750
875	2	PO/NG	mg	TAB	1750
875	2	PO/NG	mg	TAB	1750
875	2	PO/NG	mg	TAB	1750
875	2	PO/NG	mg	TAB	1750
875	2	PO/NG	mg	TAB	1750
875	2	PO/NG	mg	TAB	1750
500	4	PO	mg	CAP	2000
500	2	PO/NG	mg	TAB	1000
500	2	PO/NG	mg	TAB	1000
500	2	PO	mg	CAP	1000
875	2	PO/NG	mg	TAB	1750
875	1	PO/NG	mg	TAB	875
1000	2	PO	mg	CAP	2000
1000	2	PO	mg	CAP	2000
500	3	PO/NG	mg	CAP	1500
500	2	PO/NG	mg	SYR	1000
875	2	PO/NG	mg	TAB	1750
500	1	PO/NG	mg	TAB	500
500		PO/NG	mg	TAB	0
875	2	PO	mg	TAB	1750

875	2	PO/NG	mg	TAB	1750
875	2	PO/NG	mg	SYR	1750
875	2	PO/NG	mg	TAB	1750
875	2	PO	mg	TAB	1750
500	2	PO/NG	mg	TAB	1000
500	2	PO/NG	mg	TAB	1000
875	2	PO/NG	mg	TAB	1750
875	2	PO/NG	mg	TAB	1750
875	2	PO/NG	mg	TAB	1750
500	2	PO/NG	mg	CAP	1000
1000	2	PO/NG	mg	CAP	2000
500	3	PO	mg	CAP	1500
875	2	PO	mg	TAB	1750
500	3	PO/NG	mg	TAB	1500
1000	2	PO	mg	CAP	2000
500	2	PO/NG	mg	SYR	1000
875	2	PO/NG	mg	TAB	1750
875	2	PO	mg	TAB	1750
875	2	PO/NG	mg	TAB	1750
500	2	PO/NG	mg	TAB	1000
875	2	PO/NG	mg	TAB	1750
500	2	PO/NG	mg	TAB	1000
500	3	PO/NG	mg	TAB	1500
500	2	PO/NG	mg	TAB	1000
500	1	PO/NG	mg	TAB	500
500	1	PO/NG	mg	TAB	500
500	3	PO/NG	mg	SYR	1500
500	3	PO/NG	mg	SYR	1500
875	2	PO/NG	mg	TAB	1750
875	2	PO	mg	TAB	1750
875	2	PO	mg	TAB	1750
875	2	PO	mg	TAB	1750
875	2	PO/NG	mg	TAB	1750
875	2	PO/NG	mg	TAB	1750
500	3	PO/NG	mg	CAP	1500
500	3	PO	mg	TAB	1500
500	2	PO/NG	mg	TAB	1000
875	2	PO	mg	TAB	1750
875	2	PO/NG	mg	TAB	1750
875	2	PO/NG	mg	TAB	1750
875	2	PO/NG	mg	TAB	1750
875	2	PO/NG	mg	TAB	1750
500	2	PO/NG	mg	TAB	1000
875	2	PO/NG	mg	TAB	1750
875	2	PO/NG	mg	TAB	1750
875	1	PO/NG	mg	TAB	875

500	1	PO/NG	mg	TAB	500
1000	2	PO	mg	CAP	2000
500	3	PO/NG	mg	SYR	1500
500	2	PO	mg	TAB	1000
500	3	PO/NG	mg	CAP	1500
500	3	PO/NG	mg	CAP	1500
875	2	PO/NG	mg	TAB	1750
500	1	PO/NG	mg	TAB	500
875	2	PO/NG	mg	TAB	1750
500	3	PO	mg	TAB	1500
500	2	PO/NG	mg	TAB	1000
500	2	PO/NG	mg	TAB	1000
500	2	PO/NG	mg	TAB	1000
500	3	PO/NG	mg	SYR	1500
500	3	PO/NG	mg	SYR	1500
500	3	PO/NG	mg	SYR	1500
1000	2	PO/NG	mg	CAP	2000
1000	2	PO/NG	mg	SYR	2000
1000	2	PO/NG	mg	SYR	2000
1000	2	PO/NG	mg	SYR	2000
875	2	PO/NG	mg	TAB	1750
875	2	PO/NG	mg	TAB	1750
500	3	PO/NG	mg	CAP	1500
875	2	PO/NG	mg	TAB	1750
875	2	PO/NG	mg	TAB	1750
1000	2	PO/NG	mg	CAP	2000
875	2	PO/NG	mg	TAB	1750
500	3	PO/NG	mg	TAB	1500
875	2	PO/NG	mg	TAB	1750
500	3	PO	mg	TAB	1500
1000	2	PO/NG	mg	SYR	2000
500	2	PO/NG	mg	CAP	1000
500	2	PO/NG	mg	CAP	1000
500	2	PO/NG	mg	CAP	1000
875	2	PO/NG	mg	TAB	1750
875	2	PO/NG	mg	TAB	1750
875	2	PO/NG	mg	TAB	1750
500	1	PO/NG	mg	TAB	500
875	2	PO/NG	mg	TAB	1750
500	2	PO/NG	mg	CAP	1000
875	2	PO/NG	mg	TAB	1750
500	3	PO/NG	mg	TAB	1500
875	2	PO/NG	mg	TAB	1750
500	3	PO/NG	mg	SYR	1500
500	2	PO/NG	mg	SYR	1000
500	3	PO/NG	mg	SYR	1500

500	3	PO/NG	mg	SYR	1500
500	1	PO/NG	mg	TAB	500
500	3	PO/NG	mg	TAB	1500
875	2	PO/NG	mg	TAB	1750
875	2	PO/NG	mg	TAB	1750
875	2	PO/NG	mg	TAB	1750
875	2	PO/NG	mg	TAB	1750
500	3	PO/NG	mg	CAP	1500
500	3	PO/NG	mg	CAP	1500
1000	2	PO	mg	CAP	2000
500	3	PO/NG	mg	SYR	1500
875	2	PO/NG	mg	TAB	1750
500	2	PO/NG	mg	TAB	1000
500	3	PO/NG	mg	TAB	1500
500	3	PO/NG	mg	SYR	1500
500	3	PO/NG	mg	SYR	1500
500	2	PO/NG	mg	TAB	1000
875	2	PO/NG	mg	TAB	1750
875	2	PO/NG	mg	TAB	1750
875	2	PO/NG	mg	TAB	1750
875	2	PO/NG	mg	TAB	1750
875	2	PO/NG	mg	TAB	1750
875	2	PO/NG	mg	TAB	1750
500	3	PO/NG	mg	TAB	1500
500	2	PO/NG	mg	TAB	1000
500	2	PO/NG	mg	SYR	1000
500	3	PO/NG	mg	TAB	1500
875	2	PO/NG	mg	TAB	1750
875	2	PO/NG	mg	TAB	1750
500	3	PO/NG	mg	TAB	1500
500	2	PO/NG	mg	SYR	1000
500	3	PO/NG	mg	TAB	1500
500	4	PO/NG	mg	CAP	2000
500	2	PO	mg	CAP	1000
875	2	PO	mg	TAB	1750
500	3	PO/NG	mg	CAP	1500
875	2	PO/NG	mg	TAB	1750
875	2	PO/NG	mg	TAB	1750
500	2	PO	mg	TAB	1000
500	3	PO/NG	mg	CAP	1500
500	2	PO/NG	mg	CAP	1000
875	2	PO/NG	mg	TAB	1750
500	3	PO/NG	mg	CAP	1500
500	3	PO/NG	mg	CAP	1500
500	3	PO	mg	TAB	1500
875	2	PO	mg	TAB	1750

500	3	PO/NG	mg	SYR	1500
250	1	PO	mg	TAB	250
1000	2	PO/NG	mg	mL	2000
875	2	PO/NG	mg	TAB	1750
500	3	PO/NG	mg	TAB	1500
500	3	PO/NG	mg	CAP	1500
500	3	PO	mg	TAB	1500
500	2	PO/NG	mg	TAB	1000
875	2	PO/NG	mg	TAB	1750
500	3	PO/NG	mg	TAB	1500
875	2	PO/NG	mg	TAB	1750
500	2	PO/NG	mg	TAB	1000
875	2	PO/NG	mg	TAB	1750
875	2	PO/NG	mg	TAB	1750
875	2	PO/NG	mg	TAB	1750
500	2	PO/NG	mg	TAB	1000
875	2	PO/NG	mg	TAB	1750
875	2	PO/NG	mg	TAB	1750
500	4	PO/NG	mg	CAP	2000
500	3	PO/NG	mg	TAB	1500
875	2	PO/NG	mg	TAB	1750
875	1	PO/NG	mg	TAB	875
875	2	PO/NG	mg	TAB	1750
500	3	PO/NG	mg	CAP	1500
500	3	PO/NG	mg	TAB	1500
500	3	PO/NG	mg	TAB	1500
1000	3	PO/NG	mg	CAP	3000
500	2	PO/NG	mg	TAB	1000
500	3	PO/NG	mg	TAB	1500
500	3	PO/NG	mg	SYR	1500
500	3	PO/NG	mg	TAB	1500
875	2	PO	mg	TAB	1750
875	2	PO	mg	TAB	1750
875	2	PO/NG	mg	SYR	1750
875	3	PO/NG	mg	TAB	2625
500	1	PO	mg	TAB	500
500	1	PO	mg	TAB	500
500	2	PO/NG	mg	CAP	1000
875	2	PO/NG	mg	TAB	1750
500	3	PO/NG	mg	CAP	1500
500	3	PO/NG	mg	SYR	1500
500	2	PO/NG	mg	TAB	1000
1000	1	PO	mg	CAP	1000
1000	2	PO	mg	CAP	2000
875	2	PO	mg	TAB	1750
500	2	PO/NG	mg	TAB	1000

875	2	PO/NG	mg	TAB	1750
500	3	PO	mg	CAP	1500
875	2	PO/NG	mg	TAB	1750
500	2	PO/NG	mg	TAB	1000
500	3	PO/NG	mg	TAB	1500
1000	2	PO/NG	mg	CAP	2000
1000	2	PO/NG	mg	SYR	2000
1000	2	PO/NG	mg	mL	2000
1000	2	PO/NG	mg	CAP	2000
1000	2	PO/NG	mg	CAP	2000
500	1	PO/NG	mg	SYR	500
500	3	PO/NG	mg	CAP	1500
500	2	PO/NG	mg	TAB	1000
500	2	PO/NG	mg	TAB	1000
500	3	PO/NG	mg	SYR	1500
875	2	PO/NG	mg	TAB	1750
875	2	PO/NG	mg	TAB	1750
500	3	PO/NG	mg	SYR	1500
500	2	PO/NG	mg	CAP	1000
1000	1	PO/NG	mg	CAP	1000
875	2	PO/NG	mg	TAB	1750
1000	2	PO	mg	CAP	2000
500	2	PO	mg	TAB	1000
500	3	PO	mg	TAB	1500
500	1	PO/NG	mg	TAB	500
500	2	PO/NG	mg	TAB	1000
500	2	PO/NG	mg	SYR	1000
500	3	NG	mg	SYR	1500
500	3	NG	mg	SYR	1500
500	2	PO/NG	mg	CAP	1000
875	2	PO/NG	mg	TAB	1750
875	2	PO/NG	mg	TAB	1750
875	2	PO/NG	mg	TAB	1750
875	1	PO/NG	mg	TAB	875
500	2	PO/NG	mg	SYR	1000
875	2	PO/NG	mg	TAB	1750
875	2	PO/NG	mg	TAB	1750
500	3	PO/NG	mg	CAP	1500
875	2	PO/NG	mg	TAB	1750
500	2	PO/NG	mg	TAB	1000
875	2	PO	mg	TAB	1750
875	2	PO	mg	TAB	1750
875	2	PO	mg	TAB	1750
875	2	PO	mg	TAB	1750
500	3	PO/NG	mg	CAP	1500
875	2	PO	mg	TAB	1750

500	3	PO/NG	mg	TAB	1500
875	2	PO/NG	mg	TAB	1750
875	2	PO/NG	mg	TAB	1750
875	2	PO/NG	mg	TAB	1750
875	2	PO/NG	mg	TAB	1750
500	3	PO/NG	mg	TAB	1500
500	3	PO/NG	mg	TAB	1500
500	3	PO/NG	mg	TAB	1500
875	2	PO/NG	mg	TAB	1750
875	2	PO/NG	mg	TAB	1750
500	3	PO/NG	mg	TAB	1500
875	2	PO/NG	mg	TAB	1750
875	2	PO/NG	mg	TAB	1750
500	3	PO/NG	mg	TAB	1500
875	2	PO/NG	mg	TAB	1750
875	2	PO/NG	mg	TAB	1750
500	3	PO/NG	mg	CAP	1500
500	2	PO/NG	mg	TAB	1000
500	2	PO/NG	mg	SYR	1000
500	2	PO/NG	mg	TAB	1000
500	1	PO/NG	mg	TAB	500
875	2	PO/NG	mg	TAB	1750
500	3	PO/NG	mg	CAP	1500
500	3	PO/NG	mg	TAB	1500
500	2	PO/NG	mg	TAB	1000
500	2	PO/NG	mg	TAB	1000
500	3	PO	mg	CAP	1500
500	2	PO	mg	CAP	1000
500	2	NG	mg	SYR	1000
875	2	PO	mg	TAB	1750
875	2	PO/NG	mg	TAB	1750
500	3	PO	mg	CAP	1500
875	2	PO/NG	mg	TAB	1750
500	3	PO	mg	CAP	1500
500	3	PO	mg	CAP	1500
500	2	PO/NG	mg	TAB	1000
500	3	PO	mg	TAB	1500
875	2	PO	mg	TAB	1750
500	3	PO/NG	mg	TAB	1500
500	3	PO/NG	mg	TAB	1500
500	3	PO/NG	mg	SYR	1500
875	2	PO/NG	mg	TAB	1750
875	1	PO/NG	mg	TAB	875
1000	2	PO/NG	mg	CAP	2000
1000	2	PO/NG	mg	SYR	2000
500	3	PO/NG	mg	CAP	1500

500	2	PO/NG	mg	CAP	1000
500	2	PO/NG	mg	TAB	1000
500	2	PO/NG	mg	TAB	1000
500	2	PO/NG	mg	TAB	1000
500	2	PO/NG	mg	TAB	1000
500	3	PO/NG	mg	CAP	1500
875	2	PO/NG	mg	TAB	1750
875	2	PO/NG	mg	TAB	1750
875	2	PO/NG	mg	TAB	1750
500	3	PO/NG	mg	TAB	1500
875	2	PO/NG	mg	TAB	1750
500	3	PO/NG	mg	CAP	1500
875	2	PO/NG	mg	TAB	1750
875	2	PO/NG	mg	TAB	1750
500	3	PO/NG	mg	CAP	1500
500	2	PO	mg	CAP	1000
500	2	PO/NG	mg	SYR	1000
500	2	PO/NG	mg	TAB	1000
500	3	PO/NG	mg	TAB	1500
500	1	PO/NG	mg	SYR	500
500	2	PO/NG	mg	CAP	1000
500	3	PO/NG	mg	CAP	1500
500	3	PO	mg	CAP	1500
500	2	PO/NG	mg	TAB	1000
500	2	PO/NG	mg	SYR	1000
875	2	PO/NG	mg	TAB	1750
1000	2	PO/NG	mg	CAP	2000
875	2	PO/NG	mg	TAB	1750
875	2	PO/NG	mg	TAB	1750
875	2	PO/NG	mg	TAB	1750
875	2	PO/NG	mg	TAB	1750
875	2	PO/NG	mg	TAB	1750
500	1	PO/NG	mg	CAP	500
2000	1	PO/NG	mg	CAP	2000
1000	2	PO	mg	CAP	2000
500	2	PO/NG	mg	TAB	1000
875	2	PO/NG	mg	TAB	1750
500	3	PO/NG	mg	TAB	1500
500	3	PO/NG	mg	SYR	1500
500	2	PO/NG	mg	TAB	1000
875	2	PO/NG	mg	TAB	1750
500	3	PO/NG	mg	TAB	1500
500	3	PO/NG	mg	SYR	1500
500	3	PO/NG	mg	SYR	1500
875	2	PO/NG	mg	TAB	1750
500	3	PO/NG	mg	TAB	1500

500	2	PO/NG	mg	TAB	1000
500	3	PO/NG	mg	TAB	1500
500	3	PO/NG	mg	TAB	1500
500	3	PO/NG	mg	TAB	1500
500	2	PO/NG	mg	CAP	1000
500	1	PO/NG	mg	CAP	500
500	1	PO/NG	mg	CAP	500
500	2	PO/NG	mg	CAP	1000
1000	2	PO/NG	mg	CAP	2000
500	1	PO/NG	mg	TAB	500
875	2	PO/NG	mg	TAB	1750
500	3	PO/NG	mg	TAB	1500
875	2	PO/NG	mg	TAB	1750
500	3	PO	mg	TAB	1500
500	2	PO/NG	mg	TAB	1000
500	2	PO/NG	mg	TAB	1000
875	2	PO/NG	mg	TAB	1750
875	2	PO/NG	mg	TAB	1750
875	2	PO/NG	mg	TAB	1750
875	2	PO	mg	TAB	1750
500	2	PO/NG	mg	TAB	1000
500	2	PO/NG	mg	TAB	1000
875	2	PO/NG	mg	TAB	1750
500	3	PO/NG	mg	CAP	1500
500	3	PO	mg	TAB	1500
500	3	PO	mg	TAB	1500
